# Uncovering Molecular Biomarkers That Correlate Cognitive Decline with the Changes of Hippocampus' Gene Expression Profiles in Alzheimer's Disease

**DOI:** 10.1371/journal.pone.0010153

**Published:** 2010-04-13

**Authors:** Martín Gómez Ravetti, Osvaldo A. Rosso, Regina Berretta, Pablo Moscato

**Affiliations:** 1 Centre for Bioinformatics, Biomarker Discovery and Information-Based Medicine, The University of Newcastle, Callaghan, New South Wales, Australia; 2 Hunter Medical Research Institute, Information Based Medicine Program, John Hunter Hospital, New Lambton Heights, New South Wales, Australia; 3 Australian Research Council Centre of Excellence in Bioinformatics, Callaghan, New South Wales, Australia; 4 Instituto de Cálculo, Facultad de Ciencias Exactas y Naturales, Universidad de Buenos Aires, Ciudad Universitaria, Buenos Aires, Argentina; Universität Heidelberg, Germany

## Abstract

**Background:**

Alzheimer's disease (AD) is characterized by a neurodegenerative progression that alters cognition. On a phenotypical level, cognition is evaluated by means of the *MiniMental State Examination* (MMSE) and the post-morten examination of *Neurofibrillary Tangle* count (NFT) helps to confirm an AD diagnostic. The MMSE evaluates different aspects of cognition including orientation, short-term memory (retention and recall), attention and language. As there is a normal cognitive decline with aging, and death is the final state on which NFT can be counted, the identification of brain gene expression biomarkers from these phenotypical measures has been elusive.

**Methodology/Principal Findings:**

We have reanalysed a microarray dataset contributed in 2004 by Blalock *et al.* of 31 samples corresponding to hippocampus gene expression from 22 AD subjects of varying degree of severity and 9 controls. Instead of only relying on correlations of gene expression with the associated MMSE and NFT measures, and by using modern bioinformatics methods based on information theory and combinatorial optimization, we uncovered a 1,372-probe gene expression signature that presents a high-consensus with established markers of progression in AD. The signature reveals alterations in calcium, insulin, phosphatidylinositol and wnt-signalling. Among the most correlated gene probes with AD severity we found those linked to synaptic function, neurofilament bundle assembly and neuronal plasticity.

**Conclusions/Significance:**

A transcription factors analysis of 1,372-probe signature reveals significant associations with the EGR/KROX family of proteins, MAZ, and E2F1. The gene homologous of EGR1, zif268, Egr-1 or Zenk, together with other members of the EGR family, are consolidating a key role in the neuronal plasticity in the brain. These results indicate a degree of commonality between putative genes involved in AD and prion-induced neurodegenerative processes that warrants further investigation.

## Introduction

Gomez Ravetti and Moscato have recently shown that the abundance of five proteins, within a panel that also measured other 115 cytokines and growth factors, can be used to predict the development of clinical Alzheimer's Disease (AD) [1]. The biomarker molecular signature is composed of IL-1a, TNF-a, IL-3, EGF and G-CSF and has the same level of specificity and sensitivity as the original 18-protein signature proposed by Ray *et al.* [2] in late 2007, who introduced this important dataset in the literature. In the original work, Ray *et al.* had employed the abundance of 120 signalling proteins in plasma to obtain their 18-protein signature set. They used a training set of 83 samples to identify patients that progressed to AD in two to six years. The proposed 5-protein signature has an average of 96% accuracy in predicting clinical AD but it is still linked to the joint measurement of 120 protein abundances.

In this paper, we are revisiting the quest of finding biomarkers of AD. However, this time we aim at finding biomarkers in hippocampus tissue samples which would complement the results of the previous studies on plasma biomarkers. This study will now give a different perspective on the progression of the disease, keeping a systems biology and functional genomics approach. Towards this end, we have chosen to rely on an informative experimental design and dataset contributed by Blalock *et al.* [3]. We believe that their dataset may help us to locate, either directly or indirectly, other biomarkers of interest that could eventually be detectable in plasma.

Blalock *et al.* analysed samples from 35 patients with four different levels of AD severity: control, incipient, moderate and severe; for this paper we used only 31 samples for which information is available online. The label assigned to each sample (its “level of severity”) was decided after considering two important scores, those provided by the *MiniMental State Examination* (MMSE) and the *Neurofibrillary Tangle* count (NFT). The MMSE score is based on a questionnaire that aims at measuring the level of cognitive impairment of a patient. The questions are aimed at evaluating different aspects of cognition, such as orientation, short-term memory (retention and recall), attention and language. A normal score can range from 24 to 30, mild cognitive impairment on the interval 20 to 23, moderate AD between 10 to 19, and the rest (from 0 to 9) are all considered severe AD cases.

As previously mentioned, Blalock *et al.* [3] also used the NFT score to assign a severity label to each sample. The NFT score is a well established method for the neuropathological diagnosis of AD [4]. The score is usually based on the average counts of neurofibrilary tangles considering different regions of the brain. A NFT score is a recognised indicator of AD, nevertheless, it is not completely effective as there is evidence that NFTs were also identified in healthy aging brains [5], [6], [7], [8].

The analysis by Blalock *et al.* [3] focused on the identification of AD-related genes (ADG) and incipient ADG (IADG) using a methodology based on the correlation of the genes with NFT and MMSE scores. In turn, they identified putative biological processes and signalling pathways that are significantly present in those gene lists. Our analysis takes a different direction. While still based on the same dataset, we are attempting to map the progression of the disease, finding biomarkers linked to disease severity, by *identifying the genes associated with the divergence of the gene expression profile of a sample with the gene expression average profile of the “Control” group*. Analogously, we are interested in identifying the genes that seem to best correlate with the “convergence” to the average profile of the “AD Severe” group of samples. The difference between Blalock *et al.*'s [3] methodological approach to data analysis and ours is very important. We aim to uncover genes that correlate with the divergence of the gene expression profiles, instead of relying only on correlations with the NFT and MMSE values.

Our objective is to uncover genes which are highly correlated to the progression of the disease. With this objective in mind, we will concentrate the first part of our analysis on the two most extremely separated classes, the sets of samples that have been labelled as “Control” and those labelled “AD Severe”. This important initial decision was made based on the fact that the four classes are, in some sense, arbitrarily defined as specific thresholds for the MMSE and NFT scores that were decided *ad hoc*. Therefore, we decided to first focus on the transitional patterns that can be identified from a “normally aging” to an “AD-severe” gene expression profile in hippocampus. With this approach, we also avoid selecting genes that diverge from the normal-aged profile by causes other than AD, as we expect that the severity scale in AD has a higher probability of being correct in the “Severe AD” cases (since they have high values of NFT and low MMSE scores, clearly a joint combination highly appreciated as a disease hallmark). This approach has an additional advantage. Using this particular dataset and with focus on the effects of incorrect diagnoses, two publications indentify four possible misdiagnoses between control and incipient AD [9], [10]. In our case, the samples that have been labelled either “Incipient AD” or “Moderate AD” play the role of a “Test set”, as they are not used to select probes for establishing a molecular signature, thus avoiding misdiagnoses problems.

## Results

The results have been obtained using four steps in tandem: 1) abundance quantization of gene expression values and filtering of probes (this step is supervised by using the samples labelled either “Control” or “Severe AD”); 2) a feature selection algorithm to refine the probe selection based on numerical solution of a combinatorial optimization problem (the *(alpha,beta)-*k-Feature Set methodology); 3) a correlation analysis (that requires the computation of Jensen-Shannon divergences). Finally, a fourth step involves the pathway and Gene Ontology analysis of the results.

The first two steps only used the samples labelled either “Control” or “Severe AD”. The third step requires several procedures and uses all of the samples. We first compute an average gene expression profile for the classes “Control” and “Severe AD”. This step is followed by the computation of the *square root of the Jensen-Shannon divergence* [11] of the gene expression profile of each sample with the average profiles of the classes “Control” and “Severe AD”. Finally, we perform a correlation analysis of each gene expression profile (now across all samples) with the results of the square root of the Jensen-Shannon divergence (we do it twice, one for the “Control” and the other for the “Severe AD” case). With this information, and using state-of-the-art pathway analysis and text mining tools, as a result of our final analysis step, we provide a comprehensive list of results of the differentially regulated genes, patterns of up (down)-regulation and the pathways that seem to be implicated in the progression of AD. We refer to the Methods section for a completely reproducible and in-depth explanation of our methodology.

### Probe selection and Jensen-Shannon divergence computations based on class information

**Figure 1:**
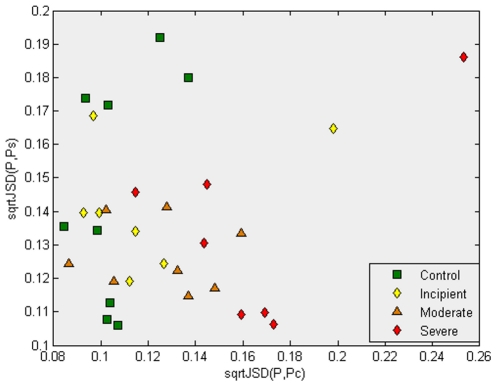
This plot illustrates that the third step of our methodology, the use of the *Jensen-Shannon divergence*, does not appear to give an interesting separation of the samples in the absence of a previous feature selection step. For this graph, all 22,215 genes were considered in the calculation of the average profile of the samples in the “Control” and “Severe AD” classes. The square root of the *Jensen-Shannon divergences* to the “Control” and “Severe AD” average profile are computed, respectively giving, for each sample, its x and y coordinates in this plot. Observe that most of the “Control” samples have values lower than 0.12, with two exceptions. This result is expected, as the probability distribution function of the “Control” class was used. However, most of the samples from AD patients (having either “Incipient AD”, “Moderate” or “Severe” labels), show a divergence with the Control average gene expression profile. Figure 2 shows the important contribution provided by the feature selection step.

We start our analysis with a baseline comparison, which we have chosen to include for illustrative purposes. Figure 1 provides an example of the importance of performing an initial probe/gene selection step. The example serves as an argument for the necessity of the first two steps of our method. We have normalized each individual gene expression profile, and we have computed the average gene expression profile for classes “Control” and “Severe AD” (following the same procedure we will use in the third step of our method, but in this case using all probes in the array).

**Figure 2:**
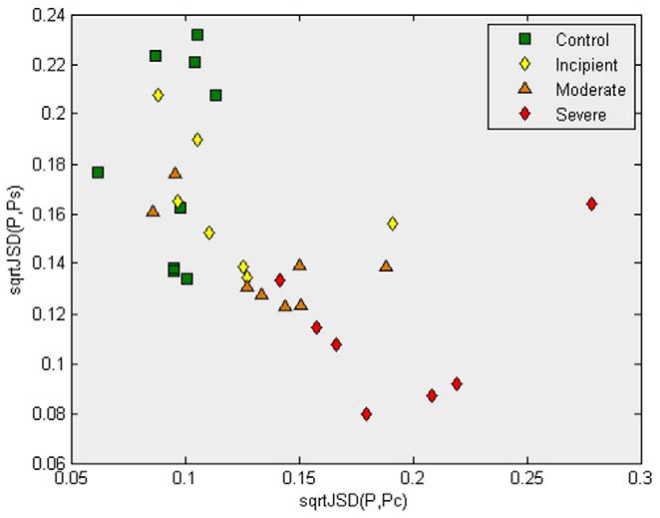
This plot illustrates that after application of the feature selection steps, followed by the computation of the gene expression profile's average profile of the samples in the “Control” and “Severe AD” classes (now on a set of 1,372 probes), the samples are now more clearly separated. Here, all “Control” samples have the square root of the *Jensen-Shannon divergences* to the average gene expression of the “Control” samples (x-coordinate) smaller than 0.12 (almost all severe AD have x-coordinates greater than 0.15). In addition to that, most samples labelled “Severe AD” are located on the same region. Both results are expected. However, it is interesting that in this (x,y)-plot most samples that are labelled “Incipient AD” or “Moderate AD” seem to “bridge” between the regions that have most of the “Control” samples and the region that have most of the “Severe AD” group. This result is interesting as no samples from “Incipient AD” nor “Moderate AD” have been used in the first three steps of our methodology. In essence, the work is a “test set” indicating that it is reasonable to expect that some genes in the genetic signature of 1,372 probes have information about a putative “progression” trend of the disease, from the “Control” to the “Severe AD” profile. In what follows, correlations across all the samples with these divergences are used as a method to try to identify those gene profiles that are most correlated with the progression from “Control” to “Severe AD”.

We have used the square root of the Jensen-Shannon divergence of a pair of samples (a pair of gene expression profiles) as our measure of “dissimilarity” between them. The square root of the Jensen-Shannon divergence quantifies the difference between two probability distribution functions (PDFs) and it is a metric (we refer the reader to the Methods section for a mathematical definition and a discussion of its properties). Figure 1 plots the divergence of each sample with the average expression profile of the classes ‘Control’ and ‘Severe AD’; *sqrtJSD(P,* 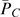 *) denotes* the square root of the Jensen-Shannon divergence between sample *P* and the average profile on the ‘Control’ class 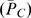. Analogously, *sqrtJSD(P ,* 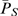 *)* denotes the square root of the Jensen-Shannon divergence between sample *P* and the average profile on the ‘Severe AD’ class 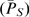. The advantage of using the probe/gene selection steps, which reduces the number of genes to the most informative ones, will be evident when we later compare Figure 1 with Figure 2. However, Figure 1 already shows some interesting patterns. For instance, we can observe that a high percentage of the samples from AD patients (having either ‘Incipient AD’, ‘Moderate’ or ‘Severe’ labels) show *sqrtJSD(P,* 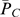 *)* values greater than 0.115, which indicates measurable divergence with the Control average gene expression profile.

Figure 2 presents the same procedure, but only after the feature selection step has significantly reduced the number of probes fom 22,215 to 1,372. We refer to the Methods section for details. In Figure 2, an arguably more coherent arrangement can be observed. As expected, the group of control samples (in green) have lower values of *sqrtJSD(P,* 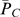 *)* and higher values of *sqrtJSD(P,* 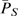 *)*. Obviously, the opposite behaviour is observed for the samples belonging to the severe cases. What cannot be expected, however, is a layout of the samples that could provide evidence of a continuous “progression” of the disease. The Figure shows that the samples of ‘Incipient AD’ are close to the control group and the ‘Moderate AD’ samples are closer to them and also link to severe AD. A priori, since those samples had not been used for probe selection, they could have been in any position in the *(sqrtJSD(P ,* 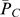 *), sqrtJSD(P,* 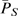 *)* plane.

**Figure 3:**
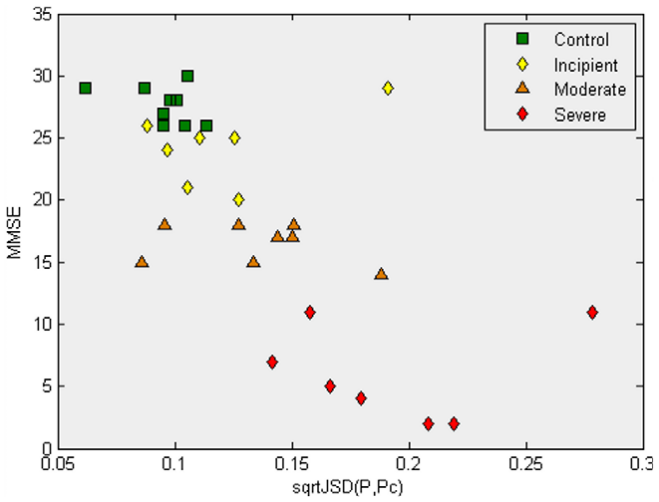
This plot shows the MMSE scores as a function of the square root of the *Jensen-Shannon divergences* to the average gene expression of the “Control” samples. ‘Incipient AD’ samples, although having a lower value for their MMSE score, still do not show a dramatic change in their x-coordinates compared to the ‘Control’ samples. ‘Moderate AD’ samples appear to be more scattered, with some of them already having a significant divergence from the ‘Control’ average profile.

Finally, Figure 3 presents the results of the MMSE score as a function of the *sqrtJSD(P ,* 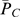 *)*, showing an inverse correlation between them. A similar situation happens between MMSE and *sqrtJSD(P,* 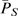 *)*, but in this case low MMSE scores correspond to low values of *sqrtJSD(P,Ps)*, giving a positive correlation. It is this interplay between positive and negative correlations that has enabled us to find interesting biomarkers. In the next subsection, we explain how these correlations were used to identify probes that “diverge from” their values in the “Control” group and “converge to” the values in the “Severe AD” group.

### Gene correlation analysis

The third step employs a correlation analysis to select the group of probes that are the most strongly correlated. Intuitively, the idea is fairly straightforward as illustrated in the following “Gedankenexperiment” (a thought experiment). Assume, for argument's sake, that the MMSE of each patient *P* is not actually phenotypical information assigned to each sample. Instead, assume that the MMSE values are the microarray probe expression of some gene. In this “thought experiment”, let *MMSE(P)* be the expression of this hypothetical gene probe on sample *P*, and *fDataset* be the set of values it has for each sample. The correlation of the sample-ordered set of values *{MMSE(P)}* with the set of sample-ordered values *{sqrtJSD(P,* 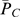 *)}* is negative, indicating that, in general, this hypothetical *MMSE* probe reduces its values as the whole gene expression profile of sample *P* diverges from the average “Control” profile (Figure 3). Analogously, there exists a positive correlation of the set of values *{MMSE(P)}* with the values of the set *{sqrtJSD(P,* 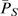 *)}*. This indicates that the values of *MMSE* tend to be reduced as the profile of sample *P* “converges to” the average profile of samples in the “Severe AD” group. We have computed these correlations for all probes in the signature, which are given in the supplementary material (File S2 sheet ‘correlation Analysis’) and are the basis for our analysis.

**Figure 4:**
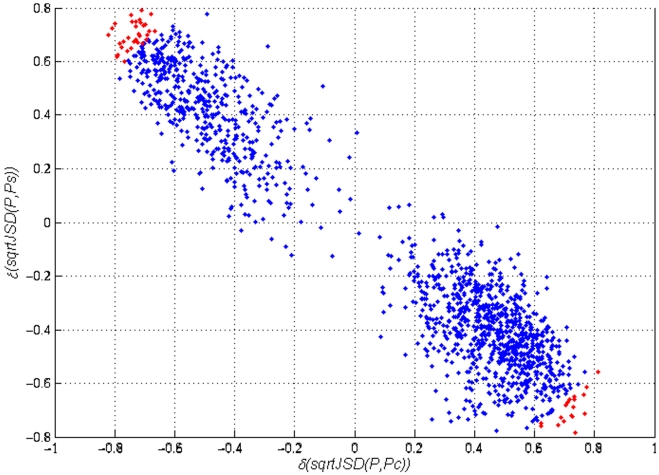
Correlation of the expression profiles of 1,372 probes (across samples) with the *sqrtJSD* of the samples of two reference groups (“Control” and “Severe AD”, represented by the average expression profile in the group). The 50 probes in red are those most distant from the origin of this system of coordinates. Those probes have expression-value variations that are correlated with the divergences of the average “Control” profile and at the same time with the “Severe AD”.

We also refer the reader to Figure 4, which presents the computed correlations. Tables 1 and 2 present the one hundred most correlated probes (in absolute values). In the supplementary material (File S2 sheet ‘correlation Analysis’), the correlation of each of the 1,372 probes that were selected by our method is given (and annotated, including Affymetrix and Stanford's Source outputs) to facilitate further analyses.

**Table 1:** For each sample, we have calculated the sample's Jensen-Shannon divergence with the average Control gene expression profile.

	Gene symbol	Probe	Spearman rank correlation
**1**	**CSF1**	**211839_s_at**	**0.79388**
**2**	**MCL1**	**214057_at**	**0.75484**
3	PSMC3IP	205956_x_at	0.74816
4	ZHX3	217367_s_at	0.74416
5	C10orf76	55662_at	0.74093
6	FCAR	211307_s_at	0.72002
7	TUBD1	210389_x_at	0.71835
8	AW974666	222365_at	0.71835
9	LRP10	201412_at	0.71079
**10**	**SERTAD2**	**202656_s_at**	**0.70679**
11	ITGB5	201125_s_at	0.7059
12	CDC2L6	212899_at	0.70412
13	RNF19A	220483_s_at	0.70367
**14**	**TTN**	**208195_at**	**0.70278**
**15**	**DHFR**	**202534_x_at**	**0.69844**
16	FYCO1	218204_s_at	0.69655
**17**	**HBEGF**	**38037_at**	**0.69388**
**18**	**ZBTB20**	**205383_s_at**	**0.69121**
19	KCNK5	219615_s_at	0.69121
20	KLHL20	204177_s_at	0.68988
21	DLG5	201681_s_at	0.68899
22	CHD2	203461_at	0.68821
23	TUG1	222244_s_at	0.68721
24	ZNF500	213641_at	0.68454
25	N58524	222332_at	0.68276
26	KIR2DL5A	211410_x_at	0.68165
27	CYBRD1	217889_s_at	0.67964
**28**	**DLG1**	**217208_s_at**	**0.67831**
**29**	**IL15**	**205992_s_at**	**0.67731**
30	RND2	214393_at	0.67508
31	TNS1	221748_s_at	0.67253
32	CTBP2	210835_s_at	0.6703
33	AL050204	213929_at	0.66852
34	YES1	202933_s_at	0.66763
35	MYBL1	213906_at	0.66719
36	No gene associated	213256_at	0.66363
37	C5orf4	48031_r_at	0.66363
**38**	**FOXO1**	**202724_s_at**	**0.66318**
**39**	**UPF1**	**211168_s_at**	**0.66096**
40	STAG3L1	221191_at	0.66007
41	SLC12A7	218066_at	0.65784
**42**	**CYP3A4**	**205999_x_at**	**0.65695**
43	KRCC1	218303_x_at	0.65562
44	P53AIP1	220402_at	0.65462
45	TLE3	212769_at	0.6535
46	ZNF669	220215_at	0.65206
**47**	**CFLAR**	**214486_x_at**	**0.65206**
**48**	**PAK4**	**203154_s_at**	**0.65028**
49	M78162	217536_x_at	0.6485
50	MMP11	203876_s_at	0.6485
51	RGS7	206290_s_at	−0.67475
52	ASTN1	213197_at	−0.67653
53	TMSB10	217733_s_at	−0.67653
54	SUPT4H1	201484_at	−0.67731
55	COX6B1	201441_at	−0.67742
56	WASF1	204165_at	−0.67742
57	RALYL	213967_at	−0.67786
58	BBS7	219688_at	−0.67875
59	SEC31A	200945_s_at	−0.68009
60	DDX1	201241_at	−0.68009
61	RP11-336K24.9	218291_at	−0.68098
62	GABBR2	209990_s_at	−0.68231
**63**	**SLC25A12**	**203340_s_at**	**−0.68454**
64	ATP5C1	205711_x_at	−0.68587
**65**	**NEFL**	**221805_at**	**−0.68632**
66	NDUFB8	201226_at	−0.68854
**67**	**OPA1**	**212214_at**	**−0.69255**
68	KPNA2	201088_at	−0.69522
**69**	**PPIA**	**211765_x_at**	**−0.69566**
70	CYP26B1	219825_at	−0.69566
71	COX7AP2	217249_x_at	−0.69878
**72**	**VSNL1**	**203798_s_at**	**−0.69878**
73	ATP6V1D	208898_at	−0.70145
74	ATP5C1	213366_x_at	−0.70234
75	NRXN1	209915_s_at	−0.7059
**76**	**PCSK2**	**204870_s_at**	**−0.70901**
**77**	**AI708767**	**211978_x_at**	**−0.71034**
78	UGCGL2	218801_at	−0.71257
79	KIAA0528	212943_at	−0.7139
**80**	**SERPINI1**	**205352_at**	**−0.71657**
81	LZTS1	219042_at	−0.71835
**82**	**NEFM**	**205113_at**	**−0.71835**
83	FRY	204072_s_at	−0.71924
84	CSPG5	205344_at	−0.72291
85	COX6A1	200925_at	−0.7277
86	COX4I1	202698_x_at	−0.73037
87	KIAA0368	212428_at	−0.73126
88	MYT1L	210016_at	−0.73304
89	PPP3CA	202457_s_at	−0.74194
**90**	**LOC100131599**	**213222_at**	**−0.74549**
91	CACNG3	206384_at	−0.75484
**92**	**PPP3R1**	**204506_at**	**−0.75573**
93	MAN1A1	221760_at	−0.75929
94	NETO2	218888_s_at	−0.76819
**95**	**LPHN1**	**219145_at**	**−0.76852**
96	CAPRIN2	218456_at	−0.76997
97	CAMK1G	215161_at	−0.77041
98	LDB2	206481_s_at	−0.7802
99	TRIM36	219736_at	−0.79622
100	LDHA	200650_s_at	−0.80245

These values are then correlated with the individual expression profiles of each probe across the set of samples samples. We list here the 100 probes that have the highest Spearman correlation (absolute value, computed over all samples) between the expression of the probe and the square root of the Jensen-Shannon divergence of the sample with the average Control gene expression profile. Rows in boldface indicate the cases for which a putative relationship exist in the published literature between the gene and AD. A probe that has a positive correlation with the square root of the Jensen-Shannon divergence with the average Control gene expression profile roughly indicates, in this case, a probe that, over all samples in the set, tends to increase its expression from their values in the “Control” group to the “Severe AD”.

**Table 2:** List of the 100 probes with the highest Spearman correlation (absolute value, computed over all samples) between the expression of the probe and the square root of the *Jensen-Shannon* divergence of all samples with the average Severe AD gene expression profile.

	Gene symbol	Probe	Spearman rank correlation
**1**	**NEFM**	**205113_at**	**0.84472**
**2**	**NRG1**	**206343_s_at**	**0.83003**
**3**	**VSNL1**	**203798_s_at**	**0.80156**
**4**	**NEFL**	**221805_at**	**0.79889**
**5**	**SLC25A12**	**203340_s_at**	**0.79666**
6	BCL11A	219497_s_at	0.79266
7	RALYL	213967_at	0.78776
**8**	**SERPINI1**	**205352_at**	**0.78242**
**9**	**ATP2B2**	**204685_s_at**	**0.78154**
10	LDB2	206481_s_at	0.7802
**11**	**ENSA**	**202596_at**	**0.77931**
12	NDUFV2	202941_at	0.77753
**13**	**KIAA0319**	**206017_at**	**0.76418**
14	ATP5C1	213366_x_at	0.7584
15	TAGLN3	204743_at	0.75617
**16**	**SV2B**	**205551_at**	**0.75484**
17	DOPEY1	213271_s_at	0.75439
18	FAR2	220615_s_at	0.75395
19	SNRK	209481_at	0.7535
20	TRIM36	219736_at	0.74994
21	NRXN1	209915_s_at	0.74772
**22**	**PKP4**	**214874_at**	**0.74461**
**23**	**CALM3**	**200622_x_at**	**0.74149**
24	PIP4K2C	218942_at	0.73971
25	CRYM	205489_at	0.73437
26	SCFD1	215548_s_at	0.73037
27	COX6A1	200925_at	0.72992
**28**	**OPA1**	**212214_at**	**0.7277**
29	ATP5C1	205711_x_at	0.72414
30	LETMD1	207170_s_at	0.71969
**31**	**PPP2R2B**	**213849_s_at**	**0.71657**
32	UQCRQ	201568_at	0.71301
**33**	**FKBP3**	**218003_s_at**	**0.71268**
34	PBX1	212148_at	0.71123
35	CACNG3	206384_at	0.71079
36	TMSB10	217733_s_at	0.70812
37	KIAA1467	213234_at	0.70812
**38**	**INA**	**204465_s_at**	**0.7059**
39	ARF5	201526_at	0.70545
**40**	**CD200**	**209582_s_at**	**0.70456**
41	CAMK1G	215161_at	0.70367
42	TUBG2	203894_at	0.70234
43	LDHA	200650_s_at	0.70189
**44**	**LOC100131599**	**213222_at**	**0.70056**
45	DIMT1L	210802_s_at	0.697
**46**	**RGS4**	**204339_s_at**	**0.69655**
47	CAMKK2	212252_at	0.69611
**48**	**BE731738**	**212661_x_at**	**0.69477**
**49**	**PPP2CA**	**208652_at**	**0.69388**
50	SRD5A1	211056_s_at	0.69388
51	DMN	212730_at	−0.68409
52	AW974666	222365_at	−0.68721
53	SLC33A1	203164_at	−0.68899
54	SYNC1	221276_s_at	−0.68954
55	ITGB5	201125_s_at	−0.69299
56	CNOT6	217970_s_at	−0.69655
57	DYNLT1	201999_s_at	−0.697
58	ZMYND8	214795_at	−0.697
59	TBL1X	213400_s_at	−0.69967
60	RND2	214393_a	−0.70378
61	LRP10	201412_at	−0.70545
62	GMPR	204187_at	−0.70768
**63**	**LTF**	**202018_s_at**	**−0.70812**
**64**	**CSNK1A1**	**208865_at**	**−0.70812**
65	NBPF12	213612_x_at	−0.70901
66	ZFP36L2	201368_at	−0.70945
67	AV712577	201305_x_at	−0.71212
68	FDFT1	208647_at	−0.71257
69	ADARB2	220648_at	−0.71301
70	CPT2	204264_at	−0.7139
71	ADD3	201753_s_at	−0.71524
72	37681	213256_at	−0.71613
73	ITGB8	205816_at	−0.71924
74	RBM19	205115_s_at	−0.71969
75	HIST1H1C	209398_at	−0.72058
76	NM_018612	220882_at	−0.73037
**77**	**CD68**	**203507_at**	**−0.73259**
78	GTF2A1L	213413_at	−0.73348
79	FAM114A1	213455_at	−0.73571
**80**	**FOXO1**	**202724_s_at**	**−0.73749**
81	C6orf145	212923_s_at	−0.73882
82	KRCC1	218303_x_at	−0.74149
**83**	**TGFBR3**	**204731_at**	**−0.74372**
84	ZHX3	217367_s_at	−0.74594
**85**	**TSPO**	**202096_s_at**	**−0.74816**
**86**	**STAT5A**	**203010_at**	**−0.74994**
87	AFF1	201924_at	−0.75039
88	RASL12	219167_at	−0.75217
89	AL359052	214927_at	−0.75528
90	ALDH3A2	202054_s_at	−0.75706
**91**	**C1S**	**208747_s_at**	**−0.76062**
**92**	**AV700298**	**217523_at**	**−0.76062**
**93**	**HBEGF**	**38037_at**	**−0.76819**
94	BG251521	213156_at	−0.77086
**95**	**ZBTB20**	**205383_s_at**	**−0.77353**
96	AL049443	215306_at	−0.78109
97	PTTG1IP	200677_at	−0.78154
98	FYCO1	218204_s_at	−0.78598
99	ATP6V0E1	214150_x_at	−0.802
**100**	**SERTAD2**	**202656_s_at**	**−0.84338**

We listed the top fifty probes with positive and negative correlation. Rows in boldface indicate the cases for which a putative relationship exist in the published literature between the gene and AD. A probe that has a positive correlation with the *square root of the Jensen-Shannon divergence* with the average Control gene expression profile roughly indicates a probe that, over all samples in the set, tends to increase its expression from their values in the “Control” group to the “Severe AD”.

As the objective is to detect the probes correlated with the progression of AD, we will select those probes with high absolute correlations values with both groups, an indication of a divergence of the average control profile together with a convergence to the severe AD profile; these correlations computed over all sample types. We need to check both groups according to their correlations to the average profile. The first group of probes we are interested in are those that have a positive correlation with the *sqrtJSD(P,* 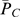 *)* and a negative correlation with *sqrtJSD(P,* 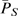 *)*. The probes in this group are those probes with under-expression in the non-disease sample but are over-expressed in the severe AD cases. The second group has the opposite behaviour, the probes' expression values have a negative correlation with *sqrtJSD(P,* 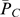 *)* and a positive correlation with *sqrtJSD(P,* 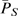 *)*. This pattern can be visualised in Figure 4, where the elliptical shape of the dispersion of the probes in this scatter plot indicates that our methodology has preserved all the significant probes for both classes and that there are no probes (after the filter) presenting a high correlation simultaneously with the control and severe AD profiles.

**Figure 5:**
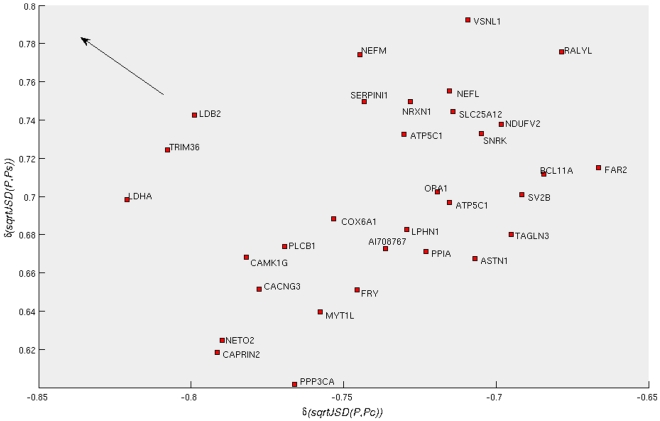
Zoom of Figure 4, identifying the most distant probes from the origin with negative correlation with the control profile, 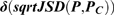 and positive correlation with the severe profile, 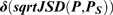.

**Figure 6:**
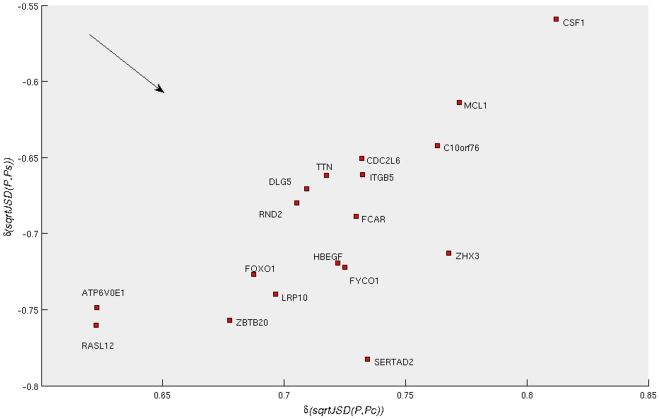
Zoom of Figure 4, identifying the most distant probes from the origin with positive correlation with the control profile, 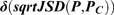 and negative correlation with the severe profile, 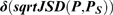.

On these values a new selection criterion is applied, as we wanted to identify the group of probes that have strong correlations to both groups in absolute value. This symmetry of our argument stems from the interest in understanding the biology of the progression of the disease. For identifying disease biomarkers we may just concentrate in finding the probes that present an upregulation trend when progressing from “Control” to “Disease”. However, here we would also like to identify those probes that become increasingly downregulated, which, in turn, would help us to identify significantly dysregulated biological pathways (as members of the pathway will be either up or downregulated). Towards this end, we rank the probes in the order given by their Euclidean distance from the origin of coordinates in Figure 4. We selected an arbitrary cut-off value of fifty probes (the selected probes are marked in red). These fifty probes are also identified by their Gene Symbols in Figures 5 and 6.

Calculating the distance of each probe to the origin, on the *sqrtJSD* system of coordinates, we further selected the 50 most distant probes and analysed their behaviour. Table 3 presents the 50 probes (corresponding to 48 genes), their correlation to each group and their distance to the origin of coordinates. File S2 sheet ‘correlation Analysis’ column ‘E - Distance’ of the supplementary material presents the distance to the origin of the 1,372 probes analysed. In Table 3, it can be seen which genes have some putative annotation that links them to AD (17 genes out of 48).

**Table 3:** The 50 genes most distant to the origin of the coordinates space 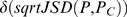
×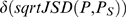.

Probe Set ID	Gene Symbol	Gene Title	*δ(sqrtJSD (P,Pc))*	*δ(sqrtJSD (P,Ps))*	Dist O	Ref (ADG)
206481_s_at	LDB2	LIM domain binding 2	−0.7988	0.7427	1.0907	
219736_at	TRIM36	tripartite motif-containing 36	−0.8077	0.7242	1.0848	
200650_s_at	LDHA	lactate dehydrogenase A	−0.8210	0.6984	1.0778	
205113_at	NEFM	neurofilament, medium polypeptide 150kDa	−0.7448	0.7742	1.0743	[379], [649], [672], [673], [674], [675], [676], [677], [678], [679], [680], [681], [682], [683], [684], [685], [686], [687], [688], [689], [690], [691], [692], [693], [694], [695], [696], [697], [698], [699], [700], [701], [702]
202656_s_at	SERTAD2	SERTA domain containing 2	0.7343	−0.7827	1.0732	
203798_s_at	VSNL1	visinin-like 1	−0.7093	0.7923	1.0634	[565], [566], [638], [641], [642], [703], [704], [705]
205352_at	SERPINI1	serpin peptidase inhibitor, clade I (neuroserpin), member 1	−0.7432	0.7496	1.0555	[706], [707], [708], [709], [710], [711], [712], [713], [714]
217367_s_at	ZHX3	zinc fingers and homeoboxes 3	0.7677	−0.7129	1.0477	
209915_s_at	NRXN1	neurexin 1	−0.7282	0.7496	1.0451	
221805_at	NEFL	neurofilament, light polypeptide 68kDa	−0.7153	0.7552	1.0402	[715], [716], [717]
213366_x_at	ATP5C1	ATP synthase, H+ transporting, mitochondrial F1 complex, gamma polypeptide 1	−0.7302	0.7327	1.0344	
203340_s_at	SLC25A12	solute carrier family 25 (mitochondrial carrier, Aralar), member 12	−0.7141	0.7444	1.0315	[718]
213967_at	RALYL	RALY RNA binding protein-like	−0.6786	0.7758	1.0307	
215161_at	CAMK1G	calcium/calmodulin-dependent protein kinase IG	−0.7819	0.6682	1.0285	
218204_s_at	FYCO1	FYVE and coiled-coil domain containing 1	0.7250	−0.7222	1.0233	
213222_at	PLCB1	phospholipase C, beta 1 (phosphoinositide-specific)	−0.7694	0.6738	1.0227	[719], [720], [721], [722], [723], [724]
200925_at	COX6A1	cytochrome c oxidase subunit VIa polypeptide 1	−0.7532	0.6883	1.0204	
38037_at	HBEGF	heparin-binding EGF-like growth factor	0.7222	−0.7194	1.0193	[725]
209481_at	SNRK	SNF related kinase	−0.7048	0.7331	1.0169	
201412_at	LRP10	low density lipoprotein receptor-related protein 10	0.6964	−0.7399	1.0161	
202941_at	NDUFV2	NADH dehydrogenase (ubiquinone) flavoprotein 2, 24kDa	−0.6984	0.7379	1.0160	
205383_s_at	ZBTB20	zinc finger and BTB domain containing 20	0.6774	−0.7569	1.0157	[726], [727]
206384_at	CACNG3	calcium channel, voltage-dependent, gamma subunit 3	−0.7778	0.6516	1.0147	
218888_s_at	NETO2	neuropilin (NRP) and tolloid (TLL)-like 2	−0.7899	0.6246	1.0070	
212214_at	OPA1	optic atrophy 1 (autosomal dominant)	−0.7194	0.7024	1.0054	[728], [729], [730], [731], [732], [733], [734], [735], [736], [737], [738], [739], [740], [741], [742]
218456_at	CAPRIN2	caprin family member 2	−0.7915	0.6186	1.0046	
211307_s_at	FCAR	Fc fragment of IgA, receptor for	0.7297	−0.6886	1.0033	
202724_s_at	FOXO1	forkhead box O1	0.6875	−0.7270	1.0006	[743], [744], [745]
219145_at	LPHN1	latrophilin 1	−0.7293	0.6826	0.9989	[746]
205711_x_at	ATP5C1	ATP synthase, H+ transporting, mitochondrial F1 complex, gamma polypeptide 1	−0.7153	0.6968	0.9986	
55662_at	C10orf76	chromosome 10 open reading frame 76	0.7632	−0.6420	0.9973	
211978_x_at	PPIA	peptidylprolyl isomerase A (cyclophilin A)	−0.7363	0.6726	0.9972	[747], [748], [749], [750]
210016_at	MYT1L	myelin transcription factor 1-like /// hypothetical protein LOC100134306	−0.7577	0.6395	0.9915	
204072_s_at	FRY	furry homolog (Drosophila)	−0.7456	0.6512	0.9899	
219497_s_at	BCL11A	B-cell CLL/lymphoma 11A (zinc finger protein)	−0.6843	0.7117	0.9873	
201125_s_at	ITGB5	integrin, beta 5	0.7323	−0.6613	0.9867	
211765_x_at	PPIA	peptidylprolyl isomerase A (cyclophilin A)	−0.7230	0.6714	0.9866	[747], [748], [749], [750]
214057_at	MCL1	Myeloid cell leukemia sequence 1 (BCL2-related)	0.7722	−0.6137	0.9864	[751]
211839_s_at	CSF1	colony stimulating factor 1 (macrophage)	0.8120	−0.5590	0.9858	[752], [753], [754], [755], [756], [757], [758], [759], [760], [761], [762], [763], [764], [765], [766], [767], [768], [769], [770], [771], [772], [773], [774], [775], [776], [777], [778], [779], [780], [781], [782]
205551_at	SV2B	synaptic vesicle glycoprotein 2B	−0.6915	0.7008	0.9846	[66]
219167_at	RASL12	RAS-like, family 12	0.6226	−0.7605	0.9828	
214393_at	RND2	Rho family GTPase 2	0.7051	−0.6799	0.9796	
212899_at	CDC2L6	cell division cycle 2-like 6 (CDK8-like)	0.7319	−0.6504	0.9791	
220615_s_at	MLSTD1	male sterility domain containing 1	−0.6665	0.7149	0.9774	
201681_s_at	DLG5	discs, large homolog 5 (Drosophila)	0.7093	−0.6706	0.9761	
208195_at	TTN	titin	0.7173	−0.6617	0.9759	[783]
202457_s_at	PPP3CA	protein phosphatase 3 (formerly 2B), catalytic subunit, alpha isoform	−0.7661	0.6016	0.9741	
214150_x_at	ATP6V0E1	ATPase, H+ transporting, lysosomal 9kDa, V0 subunit e1	0.6230	−0.7488	0.9741	
204743_at	TAGLN3	transgelin 3	−0.6952	0.6802	0.9726	
213197_at	ASTN1	astrotactin 1	−0.7069	0.6673	0.9721	

The column “Dist O” shows the Euclidean distance from the origin for each gene. If the gene has a known relation with AD (ADG), the reference's codes are display in column “Ref ADG”.

**Figure 7:**
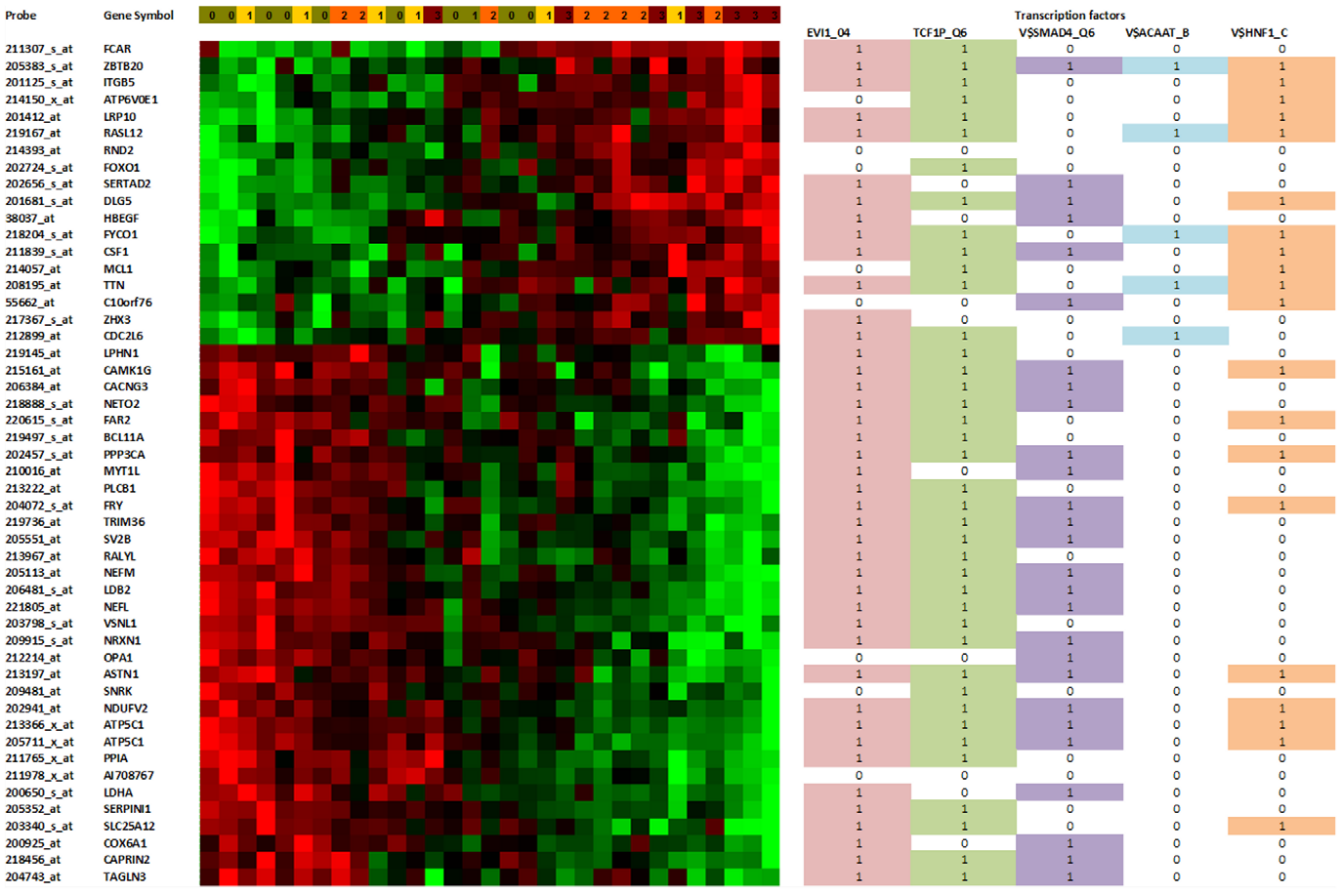
Heat map of the 50-probe signature and the transcription factors with best *p*-values, for the whole set of 50 probes and for the two groups considered. The samples and probes were sorted using the memetic algorithm given in [12], using the Euclidean distance. The transcription factors were obtained using Chang and Nevins' GATHER system to interpret genomic signatures [634]. The coloured cell and the number 1 indicate that the transcription factor has a binding motif with the gene for that row. The levels of severity as defined by Blalock *et al.* [635] are indicated in the first line: (0) Control, (1) Incipient AD, (2) Moderate AD and (3) Severe AD.

Figure 7 shows the heat map of the 50-probe signature, where the probes and patient samples are ordered by considering the similarity of their gene-expression values only. It can be observed that the Memetic Algorithm (MA), a high performance combinatorial optimization ordering method [12] for microarray datasets introduced in 2007, ordered most of the patients with or without an incipient level of AD on the left and the more severe cases on the right. When ordering the probes' gene expression, the MA perfectly sorted the groups previously described. We refer to [12], [13] for details of the MA. The supplementary material (File S2 ‘1372 norm. +heat map+GO’) presents the heat map of the 1,372 gene-probes, with samples and probes sorted by the MA.

### Transcription factors analysis of most correlated probes

**Figure 8:**
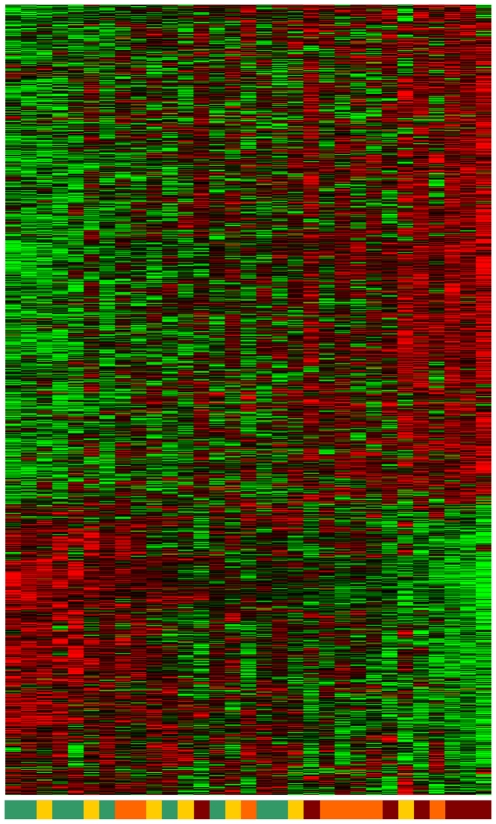
Heat map of 1,372-probe signature. The probes were sorted using the memetic algorithm but the samples remain in the same order than the 50-probe signature.

The signature of 50 probes we present in Figure 7 has 48 different genes (some probes are related to the same gene). The two repeated genes in this 50-probe list are ATP5C1 (ATP synthase, H+ transporting, mitochondrial F1 complex, gamma polypeptide 1) and PPIA (peptidylprolyl isomerase A (cyclophilin A)) [14], [15], [16], [17], a calcineurin regulatory protein. A recent study that used RT-PCR to examine tissue from 90 AD and 81 control human brains reports that cyclophilin is reduced in AD (both for females and males as compared with their gender-matched groups) [18]. We note here that the cutoff of 50 probes circumscribes the initial description a little, but most of the later discussion uses information from the whole signature to identify dysregulated pathways. Figure 8 presents the heat map of the 1,372-probe signature. The probes were sorted with the MA but the samples remain in the same position as obtained previously with the 50-probe signature.

We analysed this list of genes using GATHER [19], an online tool for annotating signatures. Forty-one genes out of fifty have a motif for EVI1 (ecotropic viral integration site 1) and thirty-nine of them have a binding motif with V$TCF1P_Q6 (TCF1: transcription factor 1, hepatic; LF-B1, hepatic nuclear factor (HNF1), albumin proximal factor). The same analysis can be done if we divide the set of genes in two groups. The first group has positive correlation with the control profile and are overexpressed in AD; the second group has a positive correlation with the severe profile, and tend towards being underexpressed in AD (see Table 3). Table 4 presents the overrepresented motifs. We note, however, that we believe that the best results to identify putative overrepresented regulatory motifs can be obtained using the whole signature of 1,372 probes, and we will present the results of this investigation after presenting the case of the most correlated probes.

**Table 4:** Binding factors related to two groups of genes.

Transcription Factors	Description	P value
*First group*		
V$EVI1_04	Ectopic viral integration site 1 encoded factor	0.00069
V$SMAD4_Q6	SMAD family member 4	0.0033
*Second Group*		
V$HNF1_C	Hepatic nuclear factor 1	0.0022
V$ACAAT_B	Avian C-type CCAAT box	0.0015

The second group has the opposite behaviour, that is, positive correlation with the severe profile.

The first group has positive correlation with the control profile.

**Figure 9:**
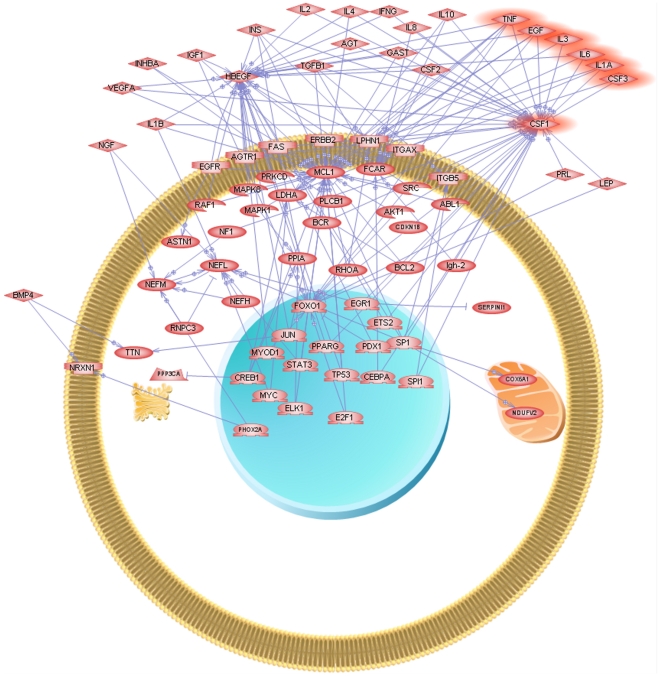
‘Common-regulators’ 50-probes’ signature. The figure was obtained using Pathway Studio [569]. The program received as input the 50-probes displayed in Fig. 7 and automatically searched all the known putative common regulators relationships. The highlighted proteins are the 5-protein signature (IL1- α, TNF-α, IL-3, EGF and GCSF) of [1]. We have also highlighted IL-6 (discussed in [1] in the context of results of classifiers that also use it) and CSF1, Colony-stimulating factor 1, (macrophage).

Another interesting pattern emerged when analysing the KEGG Pathways of the 50-probe signature using GATHER and PATHWAY Studio [20]. Using GATHER, three KEGG Pathways appear significantly represented, Amyotrophic lateral sclerosis (ALS), Oxidative phosphorylation and ATP synthesis. Using PATHWAY Studio, we automatically built the “common-regulators” diagram by selecting a filter that only considers protein interactions and binding. The resulting diagram is presented in Figure 9. As can be seen from the figure, we have chosen a circular membrane layout and our previously uncovered 5-protein signature [1] (IL1-a, TNF-a, IL-3, EGF and G-CSF) in plasma (plus IL-6) appears to have a strong relationship with CSF1 (colony stimulating factor 1 (macrophage)), the most positive correlated gene with the control profile (see Table 1). It is also worth mentioning, that CSF1 was found differentially expressed in blood of AD and Control subjects and belongs to the 18-protein signature uncovered by Ray *et al.* [2] in 2007.

### Five of the 50 most correlated probes correspond to genes already mapped to KEGGs Alzheimer's disease Pathway KEGG:05010 and together with LDHA they link to impaired metabolism and the “novel glucocorticoid hypothesis”

We have observed that five genes, which are the most correlated probes with our putative signature for disease severity, can be mapped to the AD pathway of the Kyoto Encyclopaedia of Genes KEGG:05010. They are ATP5C1, COX6A1 [21], [22], NDUFV2 [23], [24], [25], [26], [27], [28], [29], [30], PLCB1[31], [32], [33], [34], and PPP3CA (protein phosphatase 3 (formerly 2B), catalytic subunit, alpha isoform), the last one also known as Calmodulin-dependent calcineurin A subunit alpha isoform. In all cases, the probes showed a reduction of expression with AD severity, which may indicate a sign of impaired mitochondrial functions and energy uptake [35], [36].

In addition to these five, we observed the reduced expression of the glycolytic enzyme LDHA, which may also indicate another challenge for energy metabolism in these neurons. Although glucose is generally considered to be the only substrate for brain energy metabolism, moncarboxylates have also been hypotheised as alternative substrates [37]. Laughton *et al.* report segregation in the hippocampus, with LDHA present in astrocytes and not in neurons. Instead, it is pyruvate dehydrogenase that is present in neurons but not in astrocytes and as a consequence of this study they support the argument that a metabolic compartmentalization exists in the human cortex and hippocampus where lactate produced by astrocytes could be oxidized by neurons [37]. We have also observed a reduction in expression of a probe that corresponds to PDHA1 (Pyruvate dehydrogenase (lipoamide) alpha 1, 200980_s_at) with increasing AD severity. The reduction of PDH expression, and the concurrent increase in pyruvate carboxylase gene expression, was discussed by Landfield *et al.* [38], who argue that: *“These changes suggest that reduced pyruvate flux through PDH and decreased oxidative metabolism of glucose may develop early in AD. Interestingly, the inactivation of PDH is also a major pathway through which glucocorticoid activity acts to conserve glucose, and apparently, to induce insulin resistance* [*65*], [*66*] *. Thus, our data are consistent with the possibility that GC effects on this and other important target pathways in brain are enhanced in both aging and AD. If so, such alterations in glucocorticoid efficacy may have implications for AD pathogenesis as well as for the increased risk of AD associated with normal aging.”* Our results seem to indicate that LDHA might also be discussed within the extended metabolic pathways that serve as the basic framework of this novel, more complex hypothesis [38], [39], [40], [41], [42], [43], [44], [45], [46], [47], [48], [49], [50], [51], [52], [53], [54], [55].

### Four of the 50 most correlated gene probes are linked to synaptic function and neurofilament bundle assembly and also have reduced expressions with AD severity

NEFM, NRXN1, SV2B, and NEFL all have a similar pattern of reduced gene expression with AD severity. Experiments with mice depleted of the NEFL have been previously reported in the literature. Dubois *et al* state that this procedure: *“mimics the reduced NFL mRNA levels seen in amyotrophic lateral sclerosis and causes perikaryal accumulation of neurofilament proteins and axonal hypotrophy in motoneurons. NFL−/− mice was evaluated for regional brain metabolism by means of quantitative histochemical estimation of cytochrome oxidase activity.”* [56]. Mutations in the NEFL gene [56], [57], [58], [59], [60], [61], [62] and in the NEFM [63] have been linked to Charcot-Marie-Tooth disease. We will discuss the loss of expression of NRXN1 (Neurexin 1) later, when we comment on its presence in a panel of putative genes linked to prion-induced neurodegeneration [64]. However, we note here that both NRXN1 and NEFL appeared to be downregulated on a transcriptional profiling study of prion infection in mice [65].

The loss of expression of SV2B is also interesting. In 2001, Heese *et al.* [66] reported *“a new transcript of SV2B (SV2Bb) mRNA that is up-regulated at mRNA level in neurons by amyloid beta peptide (Abeta) fragment (1–42). In comparison to SV2B this new mRNA encodes for the same protein but it has an elongated 3′-untranslated region (3′UTR) that contains several AU-rich (AUR) cis-acting elements which are probably involved in posttranscriptional regulating of SV2Bb translation. In conclusion, alteration of SV2B(b) expression appears to be involved in processes of neuronal degeneration”* (see also [67]). We note that SV2B is only expressed in vesicles that undergo calcium-regulated exocytosis [68] and is a regulator of synaptotagmin 1 [69], which is a synaptic calcium sensor with a role in neurotransmitter release previously studied in AD [70], [71], [72], [73], [74], [75]. We present a number of genes related to synaptic function and neuronal plasticity which are increasingly down/up regulated later on the manuscript and on the supplementary material (File S3 Sheet ‘Synapse’).

### Analysis of the 1,372-probe signature reveals alterations in calcium and insulin signalling

Using GATHER, we have identified 32 genes in the Calcium signalling pathway http://www.genome.jp/dbget-bin/show_pathway?hsa04020 (*p*-value<0.009). They are ADCY2, **ADORA2B**, **AGTR1**, ATP2A3, ATP2B1, ATP2B2, ATP2B4, **AVPR1A**, CALM1, CALM3, *CREBBP*, GNA14, GNAS, **GRM5**, **HTR2A**, ITPR1, ITPR2, **LHCGR**, *NFATC1*, PHKA2, *PLCB1*, PLCE1, *PPP3CA*, *PPP3R1*, *PRKCB1*, **PTAFR**, SLC25A6, SLC8A2, SYK, **TBXA2R**, TNNC2, and TTN. We cannot do enough justice in this manuscript to the several different hypotheses that point at imbalances/deregulation in calcium signalling and AD pathology. Instead, we contribute to these interesting discussions with our findings of genes related to this pathway within this group of 32 genes. The gene symbols in boldface can be mapped to the KEGG Pathway hsa04080, *Neuroactive ligand-receptor interaction*; those in italics can be mapped to KEGG Pathway hsa04310, *Wnt Signalling*. Being aware of the existing interest on Wnt Signalling and AD, we went back to the list of genes present in our *(alpha,beta)-k*-feature set signature and we identified others that can also be linked to Wnt signalling, like CSNK1G3, CSNK2A2, FRAT1[76], [77], [78], [79], [80], [81], [82], [83], [84], [85], [86], [87], [88], [89], FZD5[89], [90], [91], MDFIC, PIAS4, SOX2 [92], [93], [94], [95], [96], TCF7L1/TCF3[89], [97], [98], TCF7L2/TCF4[99], [100], [101], [102], [103], [104], [105], [106], and TLE3[106], [107], [108], [109].

In addition, most of the remaining 32 genes in the *Calcium signalling pathway* can be mapped to KEGG Pathway hsa04070, *Phosphatidylinositol signalling system* (CALM1, CALM3, ITPR1, ITPR2, PLCB1, PLCE1, PRKCB1), and *Gap Junction* (ADCY2, GNA14, GNAS, GRM5, HTR2A, ITPR1, ITPR2, PLCB1, PRKCB1).

This fact suggested that we should check how many genes were mapped to these pathways. We found that *Phosphatidylinositol signalling system* was indeed the third pathway with most “hits” in our signature, and also with other 12 genes (CDIPT, CSNK1G3 PIK3C3, PIK3R1, PIK3R4, PI4KB, PIP5K1A, PIP5K1C, PIP4K2C, PTEN, SKIP and TTK) which brings the total number to 19. We have also found (CCND3, CSNK1A1, CSNK2A2, CTBP1, CTBP2, FRAT1, FZD5, PPARD, PPP2CA, PPP2R2B, RBX1, SMAD3, TBL1X, TCF7L1, TCF7L2, VANGL1) bringing the total to 22 genes. We refer the reader to the supplementary material (File S3 Sheet ‘Phosphatidylinositol signalling’) for inspection of the individual pattern of expression of all these genes.

Together with the 20 genes mapped to the Insulin signalling pathway KEGG hsa04910 (ACACA, CALM1, CALM3, EIF4E2, FOXO1A, INSR [110], [111], [112], [113], [114], [115], [116], [117], MAPK1, PDE3A, PHKA2, PIK3R1, PIK3R4, PPP1CC, PRKAR2A, PRKAR2B, PRKCI, RHEB, RHOQ, RPS6KB2, SKIP, and TSC2), our results seem to give some support to the hypothesis of altered calcium dynamics [35], [118], [119], [120], [121], [122], [123], [124], [125], [126], [127], deregulation of insulin signalling [36], [41], [113], [114], [115], [116], [128], [129], [130], [131], [132], [133], [134], [135], [136], [137], [138], [139], [140], [141], [142], [143], [144], [145], [146], [147], [148], [149], [150], [151], [152], [153], [154], [155], [156], [157], [158], [159], [160], [161], [162], [163], [164], [165] and the implication of the Wnt pathway [166], [167], [168], [169], [170], [171], [172], [173], [174], [175], [176], [177], [178], [179], [180], [181], [182], [183], [184], [185], [186], [187], [188], [189], [190], [191], [192], [193], [194], [195], [196], [197], [198], [199] in AD pathogenesis.

**Figure 10:**
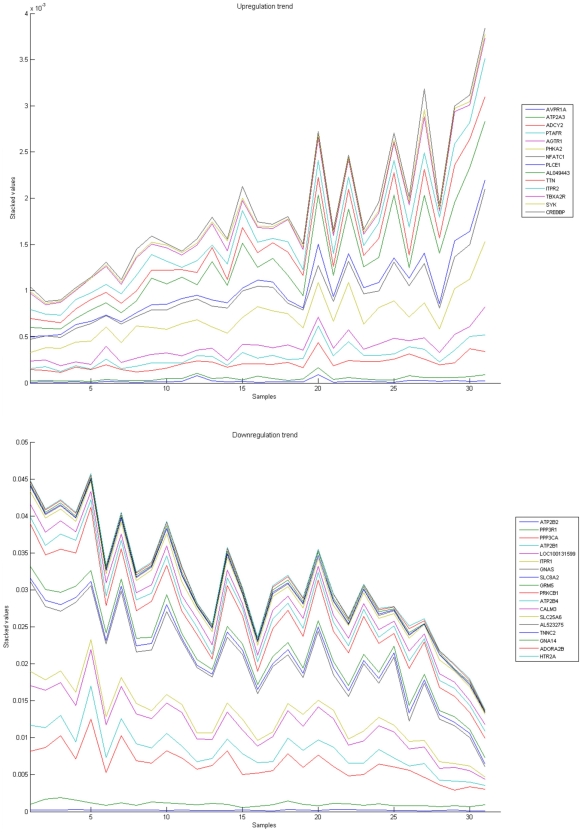
Calcium signaling pathway. The upper graph presents the stacked normalized expression values of all the probes involved in the Calcium signaling with an upregulation trend. The lower graph analyses the genes involved in the pathway with a downregulation tendency. In the supplementary material (File S3 sheet ‘Calcium signalling pathway’), the reader will find all the individual gene expression values, normalised and not normalised.

**Figure 11:**
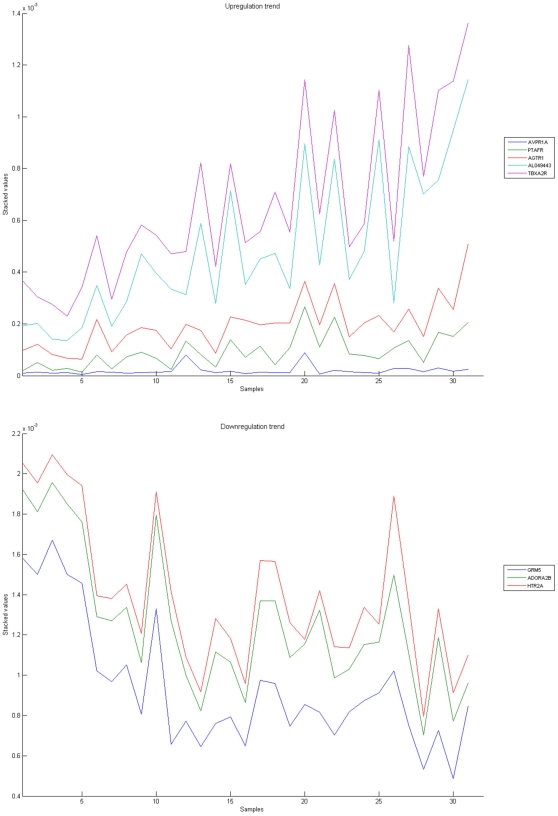
Neuroactive ligand-receptor interaction pathway. The upper graph presents the stacked normalized expression values of all the probes involved in the pathway with an upregulation trend. The lower graph analyses the genes involved in the pathway with a downregulation tendency. In the supplementary material (File S3 sheet ‘Neuroactive ligand-receptor’), the reader will find all the individual gene expression values, normalised and not normalised.

**Figure 12:**
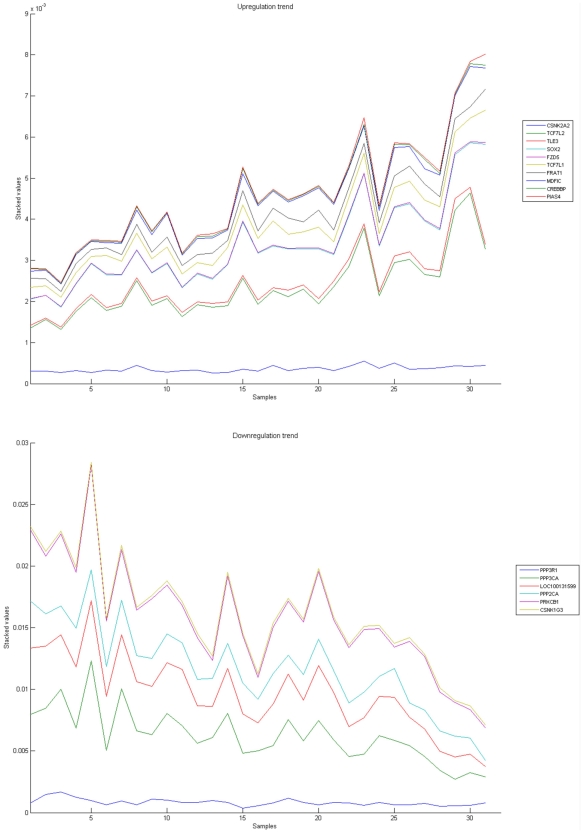
WNT signaling pathway. The upper graph presents the stacked normalized expression values of all the probes involved in the pathway with an upregulation trend. The lower graph analyses the genes involved in the pathway with a downregulation tendency. In the supplementary material (File S3 sheet ‘Wnt Signalling’), the reader will find all the individual gene expression values, normalised and not normalised.

**Figure 13:**
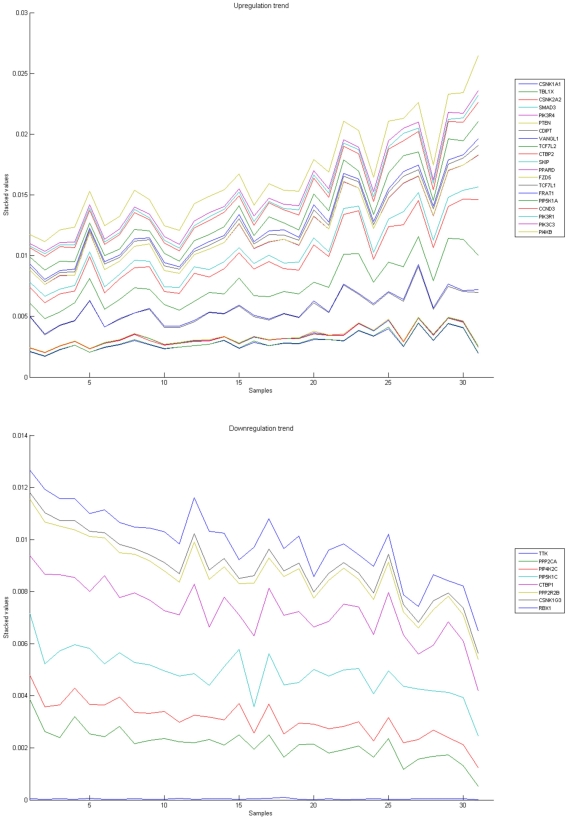
Phosphatidylinositol signaling pathway. The upper graph presents the stacked normalized expression values of all the probes involved in the pathway with an upregulation trend. The lower graph analyses the genes involved in the pathway with a downregulation tendency. In the supplementary material (File S3 sheet ‘Phosphatidylinositol signalling’), the reader will find all the individual gene expression values, normalised and not normalised.

**Figure 14:**
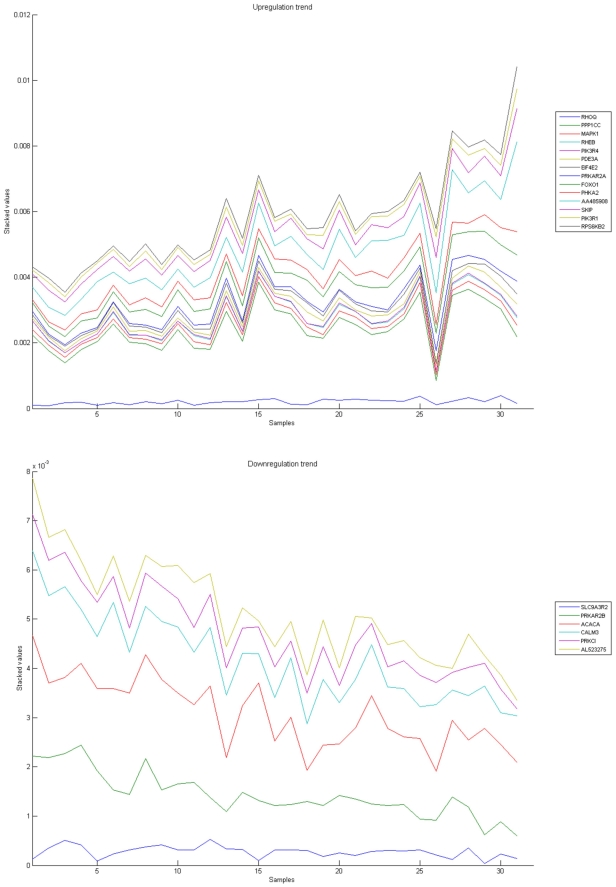
Insulin signaling pathway. The upper graph presents the stacked normalized expression values of all the probes involved in the pathway with an upregulation trend. The lower graph analyses the genes involved in the pathway with a downregulation tendency. In the supplementary material (File S3 sheet ‘Insulin signalling’), the reader will find all the individual gene expression values, normalised and not normalised.

**Figure 15:**
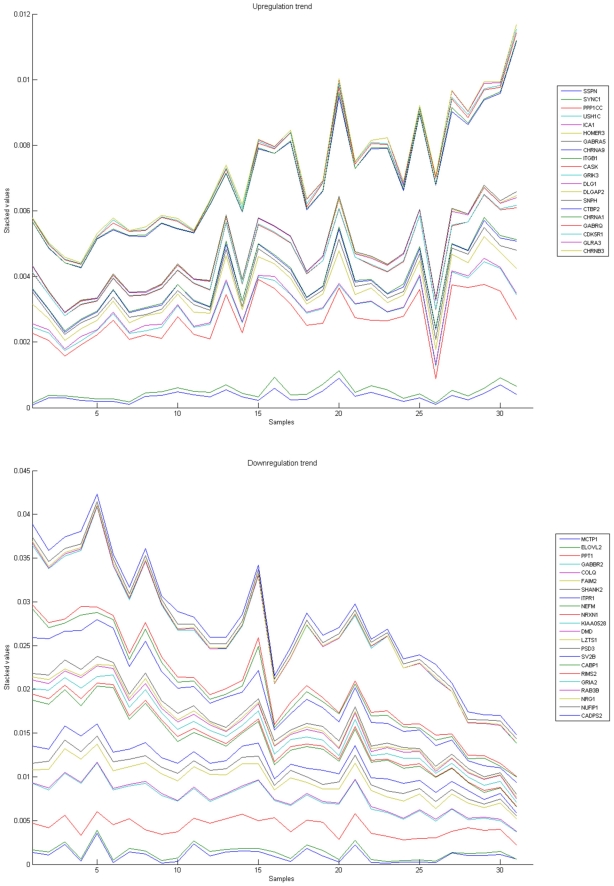
Genes related to synapse and neuronal plasticity. The upper graph presents the stacked normalized expression values of all the related probes with an upregulation trend. The lower graph analyses the genes involved with a downregulation inclination. In the supplementary material (File S3, Sheet ‘Synapse’), the reader will find all the individual gene expression values, normalised and not normalised.

Figures 10, 11, 12, 13, and 14 illustrate down(up)-regulation of genes in these signalling pathways (Calcium signalling, Neuroactive ligand receptor pathway, WNT, Phosphatidylinositol and Insulin signalling, respectively). Figure 15 shows the expression of probes corresponding to genes for which there are known associations to synaptic function and neuronal plasticity. We refer the reader to the supplementary material (File S3) for more searchable information.

### Transcription factors analysis of 1,372-probe signature reveals significant associations with the EGR/KROX family of proteins, MAZ, and E2F1

The analysis of the 1,372-probe signature indicates that they can be linked to putative transcription factors that have been previously implicated in AD and other neurodegenerative diseases. Using GATHER, we have observed that there is a strong association with motif V$KROX_Q6 (*p*-value<0.0004) with 719 out of 1294 genes in our signature; V$MAZ_Q6 (*p*-value<0.001, with 1003 genes); and V$E2F1_Q6_01 and V$E2F1_Q3_01 (with *p*-values which are smaller than 0.002 and 0.009 respectively). Of the 1294 genes associated with the 1,372 probes (by GATHER), more than half of them (656) have a motif for V$E2F1_Q6_01 and 603 have a motif for V$E2F1_Q3_01.

MAZ (MYC-associated zinc finger protein (purine-binding transcription factor)) , also known as ZF87 and Cys2His2-type zinc finger transcription factor serum amyloid A activating factor 1 [200], has been previously implicated in Alzheimer's disease [201] and as a blood biomarker in schizophrenia [202]. MAZ interacts with DCC, the receptor for netrin-1, a neuronal survival factor [203]. Deregulation of cyclin-dependent kinases and abnormal patterns of E2F1 regulation have also been linked with Alzheimer's disease [204], [205], [206], [207], [208], neurodegeneration [205], [207], [209], [210], [211], [212], [213], [214], [215], and neuronal apoptosis [216], [217], [218], [219], [220].

The involvement of the EGR/KROX (immediate early genes) family of proteins in the pathogenesis of Alzheimer's disease was first suggested in [221]. Studies of the behavioural consequences of stress have shown a link between the activation of the glucocorticoid receptor mediated response and EGR1, one of the members of this family [222]. It has been recently proposed that different members of the EGR/KROX family have different roles in learning and memory and cognitive functions [223], [224], [225], [226], [227], [228]. Mutant mice experiments showed that EGR1/KROX24 is required for the consolidation of long-term memory, while it is EGR3 the one linked to short-term memory [229], with EGR2 having perhaps other type of phenotypic characteristics not yet mapped [230]. In rat hippocampus, EGR1 decreases with aging [231]. In a recent study, it has been shown that initial playbacks of novel songs transiently increase EGR1 but that the observed response selectively habituates after repetition of the stimulus, with a different expression profile after one day [232] (see [233] and also [234] in which the homolog of NEFM, one of our biomarkers of reduced expression with increasing ‘AD severity’ called NF-M, is showed to be involved in the development and/or maturation of the oscine song control system).

We found the following connection between EGR/KROX, E2F1 and MAZ transcription factors that makes their concurrent finding notable. A recent study of microRNA signature of prion-induced neurodegeneration [64] has shown that EGR1, E2F1 and MAZ might be also implicated in the putative deregulation of immune response related genes by miRNAs via modulation of transcriptional regulators in scrapie-infected mice. We leave these findings for the next section of the manuscript where we will discuss them and present a list of common differentially expressed genes in these two neurodegenerative processes.

### The 1,372-probe signature contains a significant number of genes differentially expressed that are linked to synaptic function and neuronal plasticity

The existence of several genes among the most correlated ones (NRXN1, SV2B, NEFM, etc.,) motivated us to try to identify which genes were present in the 1,372-probe signature that are also related to synaptic function and neuronal plasticity. We have identified 42 probes that can be divided into two groups, those that seem to be increasingly downregulated with AD severity (CABP1 [235], [236], [237], [238], [239], [240], [241], [242], [243], CADPS2 [244], [245], [246], [247], [248], [249], COLQ [250], DMD [251], [252], [253], [254], [255], [256], ELOVL2 [257], FAIM2/LFG [258], [259], [260], [261], GABBR2 [262], [263], [264], [265], GRIA2/GLUR2 [266], [267], [268], [269], [270], [271], [272], [273], [274], [275], [276], [277], ITPR1 [278], [279], [280], [281], [282], [283], KIAA0528, LZTS1/FEZ1 [284], [285], NEFM, NRG1, NRXN1, NUFIP1 [286], [287], [288], PPT1 [289], [290], [291], [292], [293], [294], [295], [296], [297], [298], [299], [300], [301], PSD3, RAB3B [302], [303], [304], [305], [306], [307], [308], [309], [310], [311], [312], [313], [314], [315], [316], [317], RIMS2 [318], [319], [320], [321], SHANK2 [322], [323], [324], [325], [326], [327], [328], [329], [330], [331], [332], [333], [334], [335], [336], [337], [338], [339], [340], SV2B [68], [69], [341], [342], [343], [344], [345], [346], [347], [348], [349], [350], [351], [352], [353], [354], [355], [356], [357], [358], [359]) and those that present an upregulation pattern (CASK [360], [361], [362], CDK5R1 [363], [364], [365], [366], [367], [368], [369], [370], [371], [372], [373], [374], [375], [376], [377], [378], [379], CHRNA1, CHRNA9, CHRNB3, CTBP2, DLG1/SAP97 [380], [381], [382], [383], [384], [385], [386], [387], [388], DLGAP2, GABRA5 [389], [390], [391], [392], [393], [394], GABRQ [395], GLRA3 [396], [397], [398], GRIK3/GLUR7 [399], HOMER3 [400], ICA1 [401], ITGB1 [402], [403], MCTP1 [404], [405], PPP1CC [406], SNPH [407], [408], [409], [410], [411], [412], [413], [414], SSPN [415], SYNC1, and USH1C [416], [417], [418]). The reader can consult the supplementary material (File S2) for the individual expression patterns of these genes. If, in agreement with Klemmer *et al.* [362], consider synapses as the most complex cellular organelle, with approximately 1500 proteins interacting in an activity dependent manner, we can argue that we must be inclusive with our list of references to help other researchers map the literature of their functions. Our aim is that experts can use this information to find ways of building novel testable hypotheses of AD neuronal plasticity impairment in the hippocampus. Our approach here has been to map what is currently known, and link it with the current biomedical literature, to facilitate experts that understand processes in detail.

We have already discussed some of the increasingly downregulated genes, another important candidate for further study is NRG1 (Neuregulin 1), a gene that has already been linked to several neuronal diseases. It is a candidate for susceptibility to schizophrenia and bipolar disorder (see [419], [420], [421], [422], [423], [424], [425], [426], [427], [428], [429], [430], [431], [432], [433], [434] and references therein). There have been reported links of NRG1 with AD. BACE1 (beta-Site APP-cleaving enzyme) is necessary for the cleavage of the amyloid-beta precursor protein, and BACE1 participates in the proteolytic processing of NRG1 [435], [436], and there exists some concerns about BACE1 inhibition as a potential therapeutic intervention due to its interaction with NRG1 and potential effects on remyelination [437]. In particular, NRG1 has been reported as a possible biomarker in cerebral spinal fluid, since its levels have been reported to be significantly increased in AD. Pankonin *et al.* suggest that: *“While (NRG1) is not detected in human serum, a novel neuregulin antagonist activity was identified in human serum that could have prevented its detection. These results suggest that human neuregulin is selectively targeted from cortical neurons to white matter extracellular matrix where it exists in steady-state equilibrium with cerebral spinal fluid where it has the potential to serve as a biological marker in human neuronal disorders”* [438]. NRG1 seems to collaborate with the ERBB4 receptor, and Li *et al.* propose that together they control glutamatergic synapse maturation and plasticity [439]. A single nucleotide polymorphism in NRG1 has also been associated as a risk factor to positive symptoms of psychosis in a proportion of late-onset AD [440]. With this evidence it is clear that NGR1 [439], [441], [442], [443], [444], [445], [446], [447] as well as the whole panel presented here are excellent candidates for further studies due to their well supported role in synaptic function in health and disease states.

### Other biomarkers of interest

**Figure 16:**
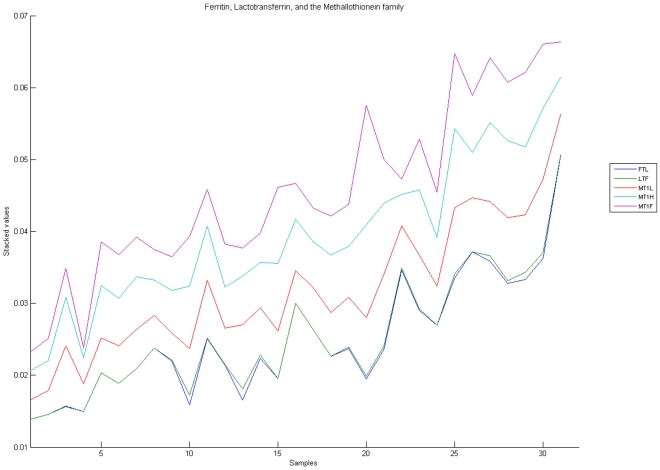
Metallothionein family. Stacked line graph of the probes related to the Metallothionein family in the 1372-probe signature.

We should also mention some other biomarkers that could be interesting for further studies, including imaging purposes, like TSPO/PBR (translocator protein (18kDa)) also known as Mitochondrial Benzodiazepine Receptor (peripheral), thus supporting its current role as a putative imaging biomarker for AD [448], [449], [450], [451], [452], [453], [454], [455], C1S (complement component 1, s subcomponent) [456], [457], [458], [459], [460], [461], FDFT1 (the squalene synthase gene), which is critical for cholesterol synthesis [462], [463], BMP4 [92], [96], [464], [465], CD68 (as marker of enhanced lysosomal activity) [450], [466], [467], [468], [469], [470], [471], [472], SERTAD2/TRIP-Br2 [473], [474], [475], LTF (Lactotransferrin) [476], [477], [478], FTL (ferritin, light polypeptide; Ferritin light chain) [479], [480], [481], [482], MTF1 (Metal-regulatory transcription factor 1) [483], [484], [485], GSTA3 (Glutathione S-transferase A3), GSTM4 (Glutathione S-transferase M4), MT1L (Metallothionein 1L (gene/pseudogene) [486] (a human-specific truncated protein which may have changed its function or suppressed it [487]), MT1H (Metallothionein 1H) [488], MT1F (Metallothionein 1F) [488], [489] (Figure 16). These last three upregulated genes need to be put in concert with other reports on methallothioneins in AD brains [490], [491], [492]. Figure 16 shows the upregulation of Lactotransferrin, FTL (ferritin, light polypeptide; Ferritin light chain), and the Metallothionein family with increasing AD severity.

**Figure 17:**
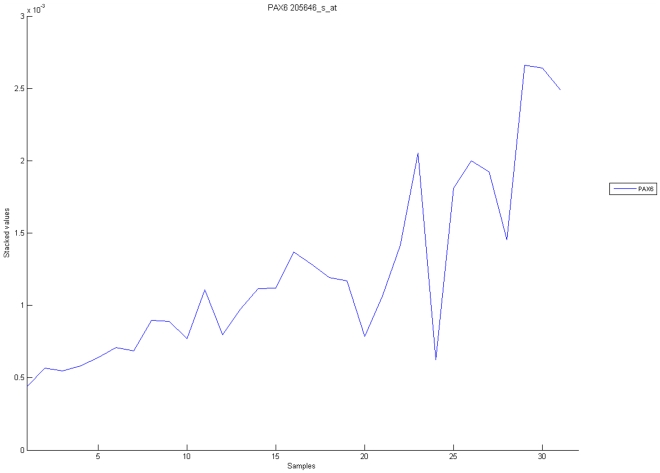
Stacked line graph of the probe expression of Ferritin Light Chain, Lactotransferrin, and the Methallothionein family, in the 1,372-probe signature, that shows an increasing upregualtion with AD severity. The expression of a PAX6 probe shows increasing upregualtion with AD severity.

**Figure 18:**
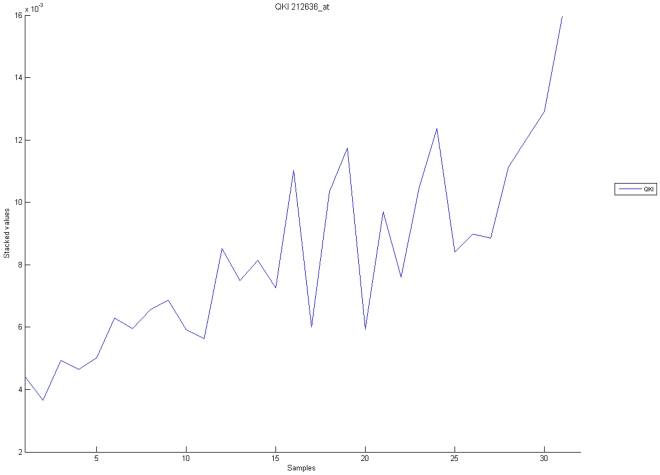
The expression of a QKI probe, like PAX6, also shows increasing upregualtion with AD severity.

Other probes which present an upregulation trend that we would like to highlight are BCL2 [493], [494], FYCO1 [495], [496], PAX6 [111], [497], [498], [499] (Figure 17), and QKI [500] (Figure 18). The increase of expression of these probes, together with SOX2, is intriguing as they are related to differentiation from stem cells and are considered critical in neurogenesis [501], [502], [503], [504], [505], [506], [507], [508], [509], [510]. Our results support the combined use of them in tracking AD progression in this tissue. In addition, we have previously mentioned the relevance of EGR1 in coordinating a large number of genes that seem to be differentially expressed in this study. EGR1 also appears with a marked upregulation in severe AD patients (we refer to the supplementary material File S2 Sheet ‘1372 norm. +heat map+GO' for its gene expression profile). We found that this link is very important, as the homologues of EGR1, zif268, Egr-1 or ZENK, together with other members of the EGR family, are consolidating a key role in the neuronal plasticity in the brain [226], [230], [511], [512], [513], [514], [515], [516], [517], [518], [519], [520], [521], [522], [523], [524], [525], [526], [527], [528], [529], [530], [531], [532], [533], [534], [535], [536], [537], [538], [539], [540], [541], [542], [543], [544], [545], [546], [547], [548], [549] and links with AD and cognitive decline progression are starting to be reported [514], [515], [550], [551], [552], [553], [554].

**Figure 19:**
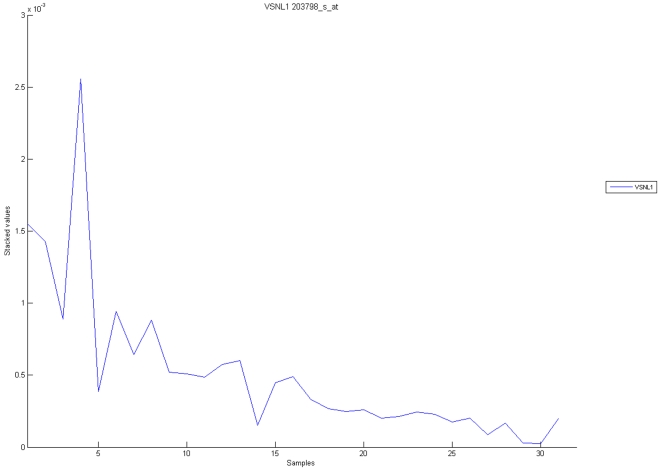
The expression of a probe for VSNL1 (Visinin-like protein-1) shows increasing downregualtion with AD severity. VSNL1, a neuronal calcium sensor that has received recent attention in AD [636], [637], [638], [639] has also been linked to model systems of schizophrenia, where it has been found upregulated in hippocampus [640]. A previous result by Schnurra *et al.* raised the possibility that the redution of VSNL1 expressing neurons indicate a selective vulnerabilty of these cells, since they observed that VSNL1 expression enhanced hyperphosphorylation of tau protein (in contrast with nontransfected or calbindin-D28K-transfected cells) [641]. In 2001, Braunewell *et al.* had already reported the reduction of VSNL1-immunoactive neurons in the temporal cortex of AD patients as compared with controls [642].

At the same time, prospective studies should encompass some other genes which appear downregulated with increasing AD severity. Top of the list is perhaps LDB2/CLIM1 (LIM domain binding 2), recently pointed as a marker (with LMO4 [555], [556]) of the control program of the development of neuronal subtype diversity of the cerebral cortex [557]. TRIM36 is another interesting candidate for further studies [558]. A gene that shares the same trend of dowregulation is CAMK1G (calcium/calmodulin-dependent protein kinase IG) [559], [560], [561], [562], [563], [564]. When analysing prefrontal cortical tissue from mice with inducible deletions of BDNF (Brain-derived neurotrofic factor), Glorioso *et al.* employed microarray gene expression profiling to show that there were alterations to early-immediate genes (including EGR1) and CAMK1G [563]. They conclude their manuscript stating that: *“while altered BDNF expression may not represent the primary disturbance in AD, changed expression of, or altered responsiveness to BDNF (and subsequently decreased SST levels) may represent a critical feature of Alzheimer's disease progression.”* VSNL1 (Visinin-like protein 1) [565], a CA++ sensor protein is also down-regulated (see Figure 19), a finding which is paralleled in the work of Youn *et al.* [566], who found similar changes in hippocampus.

## Discussion

### Putative common genes involved in Alzheimer's disease and prion-induced neurodegenerative processes

In late 2008, a paper was published in PLoS ONE, shortly after the publication of our signature for prediction of clinical symptoms of AD [1] appeared online [64]. In this other contribution, Saba *et al.* present a microRNA signature of prion induced neurodegeneration [64]. By examination of the promoter regions of putative microRNA targets, they found that some transcription factor motifs were significantly enriched, E2F-1 (*p*-value = 6.01×10^−14^), KROX (*p*-value = 9.34×10^−14^), MAZ (*p*-value = 2.23×10^−11^) and PAX6 (*p*-value = 1.76×10^−9^). Our identification of EGR1/KROX-24 and PAX-6 as upregulated with AD progression, and the identification of motif V$KROX_Q6, V$MAZ_Q6, V$E2F1_Q6_01, V$E2F1_Q3_01 as enriched in our signature were two contributing factors that motivated us to explore any further similarities that we could find.

In [64], an analysis of the predicted target genes of their microRNA signature, linked with differentially expressed genes in scrapie-infected mice [65] as well as two other publications [567], [568], led Saba *et al.* [64] to identify a network of de-regulated immune response-related genes. Additionally, they identified the putative transcription regulator genes that are targets of miRNAs similarly de-regulated. In essence, a possible hierarchy of deregulations of microRNAs, which, deregulated transcription factors that then, modify 1282 target genes. A Gene Ontology analysis also indicated that the *“data sets were found to be in the significant enrichment for genes involved in cell death, regulation of the cell cycle, nervous system development and function and cell signalling pathways.”*

**Figure 20:**
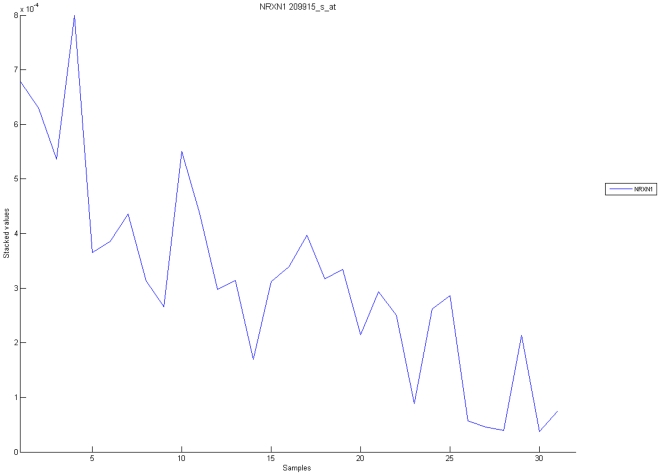
It is possible to observe that one of the probes for NRXN1 (Neurexin 1, 209915_s_at) has decreasing expression with increasing AD severity. We have found no previous evidence of a connection of NRXN1 and AD, but this gene has been previously implicated in autism [570], [571], [572], [573], [574], [575], [576], schizophrenia [577], [578], [579], [580], [581], nicotine and alcoholism dependence [582], [583], [584], and mental retardation [585].

As a consequence, we have investigated if some of the 1,282 putative target genes of the miRNA signature of prion induced neurodegeneration also appear in our lists. Of those 1,282 genes we immediately noticed that there were 9 genes listed in our list of the 50 most correlated genes (Table 3). These genes are BCL11A, CSF1, DLG5, FOXO1, HBEGF, NRXN1, SERTAD2, SNRK and ZBTB20. Two of these genes, CSF1 (colony stimulating factor 1 (macrophage)) and HBEGF (heparin-binding EGF-like growth factor) appear to be conspicuous mediators of cytokine and growth factor signalling as Figure 9 illustrates (we obtained this network using *Pathway Studio* [569] as described in the previous section), and CSF1 and HBEGF seems to be increasing with AD severity. In opposition, the probe corresponding to NRXN1 (Neurexin 1, 209915_s_at) has decreasing expression (Figure 20). Although no connection has been found between NRXN1 and AD yet, this gene has been implicated in autism [570], [571], [572], [573], [574], [575], [576], schizophrenia [577], [578], [579], [580], [581], nicotine and alcoholism dependence [582], [583], [584], and mental retardation [585]. SERTAD2 (SERTA domain containing 2), mentioned in the previous section, is also known as Transcriptional regulator interacting with the PHD-bromodomain 2, TRIP-Br2, a member of the TRIP-Br family of transcriptional regulators, required for the transduction of mitogenic signals and the execution of serum-inducible E2F-mediated cell cycle progression [473]. In our data, the probe for SERTAD2 is increasing with AD severity. It has also been reported that overexpression of SERTAD2 is sufficient to transform murine fibroblasts and promotes tumorigenesis in athymic nude mice due to the deregulation of the E2F/DP-transcriptional pathway thanks to the upregulation of the key E2F-responsive genes [474]. FOXO1 (Forkhead box O1) also appears upregulated with increasing AD severity, and has been reported as a negative regulator of EGR1 expression via the activation of the PI3K/Akt/Forkhead pathway [586]. The expression of FOXO1 is also induced by E2F1 [587]. The product of this gene has also been reported as a survival factor in deprivation-induced neuronal cell death [588], [589] (see also the review in [590]). Although FOXO1 has not been previously implicated in AD, an exception may exist. van Der Heide *et al.* describe in [591] how the Forkhead transcription factors are involved in insulin signalling. The “PI3K route” is a name given to common signal transduction cascade that links neuronal survival, synaptic plasticity (and, as a consequence, learning and memory) [592]. This “PI3K-Akt-FOXO1 mechanism” and its role in neurons warrant the current intensive investigation [593], [594], [595], [596], [597], [598], [599], [600]. From this group of 9 genes, seven of them (NRX1, SERTAD2, SNRK, HBGEF, FOXO1, CSF1, BCL11A) and QKI have been predicted to be targeted by mmu-mir128 by two or more microRNA prediction tools. We found this to be a connection that is worth exploring. Lukiw and Pogue have reported that following metal-induced reactive oxygen species production (by iron and aluminium-sulfate at nanomolar concentrations) upregulates miR-128 in human neural cells in primary culture [601]. They also report that, together with miR-9, mir-125a, mir-128 is upregulated in AD brain. In the previously cited reference Lukiw reported that: *“miR-9, miR-124a, miR-125b, miR-128, miR-132 and miR-219 are abundantly represented in fetal hippocampus, are differentially regulated in aged brain, and an alteration in specific micro-RNA complexity occurs in Alzheimer hippocampus.”*

**Figure 21:**
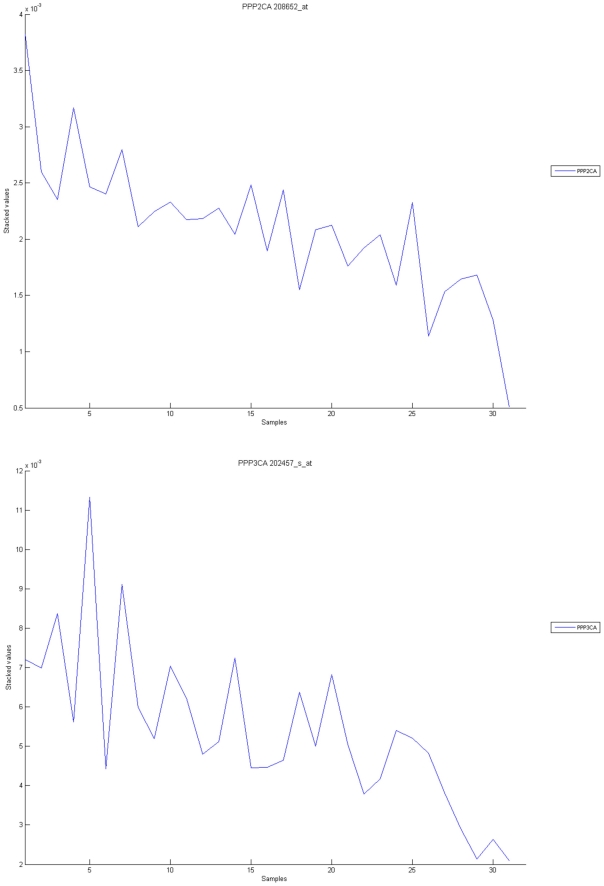
The expression of two probes for PPP2CA (Protein phosphatase 2 (formerly 2A), catalytic subunit, alpha isoform,) and PPP3CA (Protein phosphatase 3 (formerly 2B), catalytic subunit, alpha isoform, Calcineurin A1) show increasing downregualtion with AD severity. A similar plot exists for PPP3R1 (protein phosphatase 3 (formerly 2B), regulatory subunit B, alpha isoform, Calcineurin subunit B type 1). This result supports a role for downregulation of PPP2CA, PPP3CA in AD pathology [643], [644], [645], [646], [647], [648], [649], [650], [651], [652], [653], [654], [655], [656], [657], [658], [659], [660], [661], [662], [663], [664], [665], [666], [667], [668], [669], [670], [671].

The expression of probes corresponding to PP2A and PP2B catalytic subunits (i.e. PPP2CA, Protein phosphatase 2 (formerly 2A), catalytic subunit, alpha isoform, and PPP3CA, Protein phosphatase 3 (formerly 2B), catalytic subunit, alpha isoform, Calcineurin A1) shows increasing downregualtion with the progression of AD., see Figure 21. This finding supports a role for downregulation of PPP2CA, PPP3CA in AD pathology [619]–[647].

Finally, in addition to the presence of hyperphosphorylated tau, the accumulation of Amyloid-beta (Abeta) peptide in brain tissue is a hallmark of AD [602]. The identification of the genes involved in the proteolytic processing of APP (beta-amyloid precursor protein), which in turn produces Abeta, is a subject of intense research. Researchers are currently looking at the alterations of APP cellular localization and endocytic trafficking as one mechanism that can modify the processing of APP to Abeta. LRPs are known to regulate APP's endocytic trafficking [603], [604], [605], [606], and seem to be a hub of a number of mounting evidences on processes that link to cholesterol metabolism and atherosclerosis [607]. In our selected panel of 50 proteins we have one member of this family, LRP10 (low density lipoprotein receptor-related protein 10), as one of the most correlated gene expression profiles. In our list of 1372 gene probe signature we also have another member of this family, LRP1B (low density lipoprotein-related protein 1B (deleted in tumors))[608], While LRP10 appears to be positively upregulated with cognitive decline an inverse relationship is observed for LRP1B.

LRPs are also known to linked to APP via a mechanism that involves the alternative splicing of APBB3/Fe65L2 [609], [610], [611]. Tanahashi and Tabira have proposed that the splicing of APBB3/Fe65L2 alters the ability to bind with APP and low-density-lipoprotein-receptor-related protein. They propose that the secretion of beta-amyloid peptide Abeta40 and Abeta42 is increased following the overexpression of APBB3, but there are no visible changes of half-life and maturation of APP, or the secretion of secreted APP [612]. In our dataset, we observe APBB3 expression being upregulated with the increasing cognitive decline, following the same pattern of LRP10.

Polymorphisms on these genes have previously been linked to AD. Tanahashi, Asada and Tabira have reported an association between a polymorphism in APBB3/Fe65L2 and early-onset AD [612] (the link between APBB3 and AD is being increasingly explored, we refer to [613], [614], [615], [616] for further references). Using 500K SNP microarray technology, Poduslo, Huang and Spiro have identified haplotypes in LRP1B as significant for successful aging without cognitive decline in a study involving individuals that were 85 years old or older, had MMSE scores greater than 26, no history of dementia in their families, and no major illnesses (i.e. no cardiovascular problems, diabetes, obesity, or major cancer diseases) and most of them had normal cholesterol levels. Their genome-wide association screening compared these individuals with those that have late-onset AD [617]. Poduslo et al. have suggested that if the decreased production of Abeta42 in successful aging is due to the haplotypes they describe, then LRP1B may be a new target for treatment of AD [608], [617], Taken together these results indicate that integrative bioinformatics analytic approaches will be needed to elicit the interactome of LRPs and their role in AD.

### Conclusions

This re-analysis of the microarray dataset hippocampal gene expression contributed by Blalock *et al.* has shown that there exist a relatively large number of probes (1,372) that present a clear pattern of either up or down regulation with increasing AD severity. The signature reveals alterations in calcium, insulin, phosphatidylinositol and Wnt-signalling. Among the group of most correlated gene probes with AD severity we found some linked to synaptic function, neurofilament bundle assembly, neuronal plasticity and inflammation.

A transcription factors analysis of 1,372-probe gene expression signature reveals significant associations with the EGR/KROX family of proteins, MAZ, and E2F1. The gene homologous of EGR1, zif268, Egr-1 or ZENK, together with other members of the EGR family, are consolidating as key players in short and long-term memory and neuronal plasticity in the brain. We have also uncovered a large consensus of this gene expression signature with current genes putatively involved in AD progression. Our results also indicate a degree of commonality between putative genes involved in AD and prion-induced neurodegenerative processes that warrants further investigation.

## Materials and Methods

### Dataset

In this contribution, we have used a MIAME compliant, Affymetrix gene expression dataset that is public available and was contributed by Blalock et al [3] in 2004. We thank the authors of that publication for making this useful dataset available to the research community at large allowing further exploration and reanalysis.

The dataset is available from GEO Dataset Browser, accession number GDS1297 (http://www.ncbi.nlm.nih.gov/geo/query/acc.cgi?acc=GSE1297). The Affymetrix human GeneChip, HG-U133A, containing 22,283 targets was used. The dataset is de-identified and the methods for disease classification, based on MMSE and NFT scores, are described in full detail by Blalock et al. in Ref. [3].

The hippocampal samples used by Blalock et al. were obtained from the autopsy of 31 subjects through the Brain Bank of the University of Kentucky Alzheimer's Disease Research Center (ADRC), Sanders-Brown Center on Aging, University of Kentucky. The ADRC was established in 1985 and in operation since 1989 a pool of research volunteers that have agreed in principle to be research participants. Participants were asked questions based on NINCDS/ADRDA criteria [618] to establish their physical and mental condition to determine if their were eligible for the study. When a mutual agreement existed, the individuals were visted in their homes to review and sign the informed-consent document (which was approved by the University of Kentucky Institutional Review Board). Participants also signed a donor card, and the visit also aimed to establish their baseline mental-status testing. Elegibility for the purpose of the study included having a Mini-Mental State Exam score above 24 [619], passing a series of cognitive tests, and a previous history of absence of neurological disease [620], as well as neither substance abuse nor major psychiatric illnesses. All eligible volunteers were 60 years of age or older and satisfactorily performed normal activities of daily living. The Wechsler Adult Intelligence Scale (Vocabulary) was also applied to exclude significant other medical diseases that could affect cognition and elegible participants must had no previous history of head injury with loss of consciousness.

The research participants that were deemed eligible also signed a form (in addition to the consent document) indicating their agreement to donate their brain to the Sanders-Brown Center on Aging. A full description of the methods used can be found in Brain Donation in Normal Aging Procedures, Motivations, and Donor Characteristics from the Biologically Resilient Adults in Neurological Studies (BRAiNS) Project [621].

Blalock et al. [3] categorized the samples in four groups, with a labelling that indicates different “levels of severity”. These labels were decided based on the *MiniMental State Examination* (MMSE) and the *Neurofibrillary Tangle* count (NFT) of each sample [622]. Samples are then separated in the types ‘Control’, ‘Incipient AD’, ‘Moderate AD’ and ‘Severe AD’. Table 1 of Blalock et al. shows the mean values of MMSE and NFT for each one of these groups. In addition, they give the mean Braak stage [623], [624], [625] for each one of the groups (2.1 for ‘Control’, 5 for ‘Incipient’, 5.6 for ‘Moderate’ and 5.9 for ‘Severe’). We are grateful to Dr. Blalock who has kindly given us these values of the Braak stage for each sample in the dataset. Together with the individual values of MMSE, NFT, the Braak stage of each sample is included in the Supplementary Material (File S2 sheet ‘Braak’) section of this publication.

### Methodology

Our analysis method consisted of four steps: abundance quantization and filtering of probes; a feature selection algorithm to refine the probe selection; a Jensen-Shannon divergence computation; and finally, a correlation analysis. Each of these steps is described below.

As mentioned in the Results section, we only used the samples labelled as “Control” or “Severe AD” for feature selection, thus we have a two-class probe/gene selection task. We did not use the samples labelled as “Incipient AD” or “Moderate AD” for the probe selection steps. Those samples were only used in the final step, at the time of computing the correlation of the gene profile, across all samples, with the Jensen-Shannon divergences computed for the “Control” and “Severe” classes as explained later in this section.

For the first step, the quantization of the expression values, as well as for the initial data pruning, we used Fayyad and Irani's algorithm [626]. The heuristic algorithm minimises the feature-class entropy and discards genes according to the Minimum Description Length principle. The application of Fayyad and Irani's algorithm not only filters several thousand genes, it also provides thresholds for each probe remaining in the dataset. These quantized values of gene expression leave us with an instance of a combinatorial optimization problem, the *(α, β)-k-Feature Set problem* [13], [627], [628].

The *(α, β)-k-Feature Set problem* is a combinatorial optimisation problem introduced by Cotta, Sloper and Moscato[628] in 2004 to address the problem of feature selection in high-dimensional datasets. We solve an instance of this problem numerically using an integer programming formulation. This approach has been previously employed to obtain molecular biomarker signatures in Alzheimer's Disease [1], [629], models of Parkinson disease [630], prostate cancer [631], electrode selection in EEGs [632], and elsewhere. To obtain mathematically proven optimal solutions of the integer programming formulation, the CPLEX commercial optimization solver was used. As in previous contributions of our group, we found gene expression signatures corresponding to values of *α* maximum and *β* maximal [1], [13], [627], [628], [633]. We refer the reader to these previous contributions for a detailed explanation of the methodology.

At this point, we have a selection of 1,372 probes, a set which we denote as Ω. For each sample *m* and probe 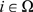, let *f_im_* be its expression value. We now define a probability distribution function (PDF) for each sample. For sample, *m* its PDF 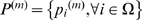, is given by
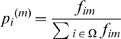
We can now compute an average PDF profile for samples in the “Control” and “Severe AD” groups, denoted by 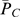 and 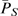 respectively. Let *C* and *S* be the set of samples with the labels “Control” and “Severe AD” respectively. The average profile 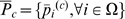, is then:
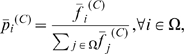
where
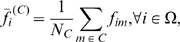
where 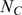 represents the number of samples in class *C*. 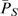 is analogously defined.

The *Jensen-Shannon divergence* between two sample PDFs, i.e. samples *l* and *k* (*P^(l)^* and *P^(k)^*) is defined as
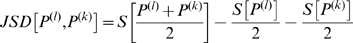
where 
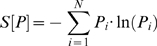

*S*[*P*] is the *Shannon Entropy* for a specific PDF sample with *N* states. It is well known that the square root of the JSD (*sqrtJSD*) is a metric, which means that for a given set of PDFs the following four properties are satisfied:

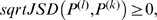

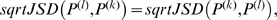

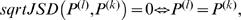

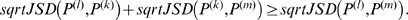

Once the *sqrtJSD* between each patient and the two average profiles (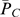 and 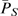) has been computed, the genes most correlated with these metrics can be uncovered. We used the Spearman rank correlation, which is a well-known non-parametric method, and can thus be used even when the data does not satisfy assumptions about normality, homoscedasticity and linearity.

### Supplementary Material

Supplementary ‘File S1’ provides a glossary of each gene referenced in this paper including synoms and refrences to iHOP (http://www.ihop-net.org/).

The results referenced in this manuscript are provided in supplementary ‘File S2’ and ‘File S3’ in Microsoft Excel format.

## Supporting Information

File S1 IHop Glossary of Genes.
(0.15 MB DOC)

File S2 Supplementary Data 1.
(4.10 MB XLS)

File S3 Supplementary Data 2.
(1.26 MB XLS)
