# Supplementary material for: Uncovering Molecular Biomarkers That Correlate Cognitive Decline with the Changes of Hippocampus' Gene Expression Profiles in Alzheimer's Disease
Source: PLoS One. 2010 Apr 13;5(4):e10153. doi: 10.1371/journal.pone.0010153 (PMC2854141; doi:10.1371/journal.pone.0010153)
Supplement: File S1 — IHop Glossary of Genes. (0.15 MB DOC) [file pone.0010153.s001.doc]

**Glossary of Gene Names and Aliases used and/or discussed in this paper**

(as provided by iHOP – Information hyperlinked over proteins)

**ACACA**

**Name:** acetyl-Coenzyme A carboxylase alpha

**Aliases:** ACAC, ACC, ACC1, ACCA, ACC-alpha, Acetyl-CoA carboxylase 1

<http://www.ihop-net.org/UniPub/iHOP/mgi/86174.html>

**ADCY2**

**Name:** adenylate cyclase 2 (brain)

**Aliases:** AC2, Adenylate cyclase type 2, Adenylate cyclase type II, Adenylyl cyclase 2, ATP pyrophosphate-lyase 2, FLJ16822, FLJ45092, HBAC2, KIAA1060, MGC133314

<http://www.ihop-net.org/UniPub/iHOP/mgi/86248.html>

**ADORA2B**

**Name:** adenosine A2b receptor

**Aliases:** Adenosine receptor A2b

<http://www.ihop-net.org/UniPub/iHOP/mgi/86274.html>

**AGTR1**

**Name:** angiotensin II receptor, type 1

**Aliases:** AG2S, AGTR1A, AGTR1B, AT1, AT1AR, AT1B, AT1BR, AT1R, AT2R1, AT2R1A, AT2R1B, HAT1R, Type-1 angiotensin II receptor

<http://www.ihop-net.org/UniPub/iHOP/mgi/86320.html>

**ATP2A3**

**Name:** ATPase, Ca++ transporting, ubiquitous

**Aliases:** Calcium pump 3, Sarcoplasmic/endoplasmic reticulum calcium ATPase 3, SERCA3, SR Ca(2+)-ATPase 3

<http://www.ihop-net.org/UniPub/iHOP/mgi/86613.html>

**ATP2B1**

**Name:** ATPase, Ca++ transporting, plasma membrane 1

**Aliases:** Plasma membrane calcium ATPase isoform 1, Plasma membrane calcium pump isoform 1, Plasma membrane calcium-transporting ATPase 1, PMCA1, PMCA1kb

<http://www.ihop-net.org/UniPub/iHOP/mgi/86614.html>

**ATP2B2**

**Name:** ATPase, Ca++ transporting, plasma membrane 2

**Aliases:** Plasma membrane calcium ATPase isoform 2, Plasma membrane calcium pump isoform 2, Plasma membrane calcium-transporting ATPase 2, PMCA2, PMCA2a, PMCA2i

<http://www.ihop-net.org/UniPub/iHOP/mgi/86615.html>

**ATP2B4**

**Name:** ATPase, Ca++ transporting, plasma membrane 4

**Aliases:** ATP2B2, DKFZp686G08106, DKFZp686M088, Matrix-remodeling-associated protein 1, MXRA1, Plasma membrane calcium ATPase isoform 4, Plasma membrane calcium pump isoform 4, Plasma membrane calcium-transporting ATPase 4, PMCA4, PMCA4b, PMCA4x

<http://www.ihop-net.org/UniPub/iHOP/mgi/86617.html>

**AVPR2**

**Name:** arginine vasopressin receptor 2

**Aliases:** ADHR, Antidiuretic hormone receptor, AVPR V2, DI1, DIR, DIR3, MGC126533, MGC138386, NDI, Renal-type arginine vasopressin receptor, V2R, Vasopressin V2 receptor

<http://www.ihop-net.org/UniPub/iHOP/mgi/86669.html>

**ATP5C1**

**Name:** ATP synthase, H+ transporting, mitochondrial F1 complex, gamma polypeptide 1

**Aliases:** ATP5C, ATP5CL1, ATP synthase subunit gamma, mitochondrial, F-ATPase gamma subunit

<http://www.ihop-net.org/UniPub/iHOP/mgi/86631.html>

**ATP8A2**

**Name:** ATPase, aminophospholipid transporter-like, class I, type 8A, member 2

**Aliases:** ATP, ATPase class I type 8A member 2, ATPIB, DKFZp434B1913, IB, ML-1

<http://www.ihop-net.org/UniPub/iHOP/mgi/99616.html>

**BACE1**

**Name:** beta-site APP-cleaving enzyme 1

**Aliases:** ASP2, Asp 2, Aspartyl protease 2, BACE, Beta-secretase 1, Beta-site amyloid precursor protein cleaving enzyme 1, Beta-site APP cleaving enzyme 1, FLJ90568, HSPC104, KIAA1149, Memapsin-2, Membrane-associated aspartic protease 2

<http://www.ihop-net.org/UniPub/iHOP/mgi/97000.html>

**BCL11A**

**Name:** B-cell CLL/lymphoma 11A (zinc finger protein)

**Aliases:** B-cell CLL/lymphoma 11A, B-cell lymphoma/leukemia 11A, BCL11A-L, BCL11A-S, BCL11A-XL, COUP-TF-interacting protein 1, CTIP1, Ecotropic viral integration site 9 protein homolog, EVI9, EVI-9, FLJ10173, FLJ34997, HBFQTL5, KIAA1809, ZNF856

<http://www.ihop-net.org/UniPub/iHOP/mgi/99635.html>

**BDNF**

**Name:** brain-derived neurotrophic factor

**Aliases:** Abrineurin, Brain-derived neurotrophic factor, obsessive-compulsive disorder 1, OCD1

<http://www.ihop-net.org/UniPub/iHOP/mgi/86737.html>

**BMP4**

**Name:** bone morphogenetic protein 4

**Aliases:** BMP2B, BMP-2B, BMP2B1, BMP-4, Bone morphogenetic protein 4, DVR4, MCOPS6, OFC11, ZYME

<http://www.ihop-net.org/UniPub/iHOP/mgi/86761.html>

**C1S**

**Name:** complement component 1, s subcomponent

**Aliases**: Basic proline-rich peptide IB-1, C1 esterase, Complement C1s subcomponent

<http://www.ihop-net.org/UniPub/iHOP/mgi/86819.html>

**CABP1**

**Name:** calcium binding protein 1

**Aliases:** CaBP1, Calbrain, CALBRAIN, Calcium-binding protein 1, HCALB_BR

<http://www.ihop-net.org/UniPub/iHOP/mgi/94688.html>

**CADPS2**

**Name:** Ca++-dependent secretion activator 2

**Aliases:** Calcium-dependent activator protein for secretion 2, Calcium-dependent secretion activator 2, CAPS2, CAPS-2, FLJ40851, KIAA1591

<http://www.ihop-net.org/UniPub/iHOP/mgi/105093.html>

**CALM1**

**Name:** calmodulin 1 (phosphorylase kinase, delta)

**Aliases:** CALML2, CAMI, DD132, PHKD

<http://www.ihop-net.org/UniPub/iHOP/mgi/86894.html>

**CALM3**

**Name:** calmodulin 3 (phosphorylase kinase, delta)

**Aliases:** PHKD, PHKD3

<http://www.ihop-net.org/UniPub/iHOP/mgi/86901.html>

**CAMK1G**

**Name:** calcium/calmodulin-dependent protein kinase IG

**Aliases:** Calcium/calmodulin-dependent protein kinase type 1G, CaMKIG, CaMKI gamma, CaMKI-gamma, CaM-KI gamma, CaM kinase IG, CaM kinase I gamma, CaMK-like CREB kinase III, CLICKIII, CLICK III, dJ272L16.1, VWS1

<http://www.ihop-net.org/UniPub/iHOP/mgi/101340.html>

**CASK**

**Name:** calcium/calmodulin-dependent serine protein kinase (MAGUK family)

**Aliases:** CAGH39, Calcium/calmodulin-dependent serine protein kinase, CMG, FGS4, FLJ22219, FLJ31914, hCASK, LIN2, Lin-2 homolog, MICPCH, Peripheral plasma membrane protein CASK, TNRC8

<http://www.ihop-net.org/UniPub/iHOP/mgi/93965.html>

**CCND3**

**Name:** cyclin D3

**Aliases:** G1/S-specific cyclin-D3

<http://www.ihop-net.org/UniPub/iHOP/mgi/86979.html>

**CD68**

**Name:** CD68 molecule

**Aliases:** DKFZp686M18236, Gp110, GP110, macrosialin, Macrosialin, SCARD1

<http://www.ihop-net.org/UniPub/iHOP/mgi/87049.html>

**CDIPT**

**Name:** CDP-diacylglycerol--inositol 3-phosphatidyltransferase (phosphatidylinositol synthase)

**Aliases:** CDP-diacylglycerol--inositol 3-phosphatidyltransferase, MGC1328, Phosphatidylinositol synthase, PIS, PIS1, PI synthase, PtdIns synthase

<http://www.ihop-net.org/UniPub/iHOP/mgi/95526.html>

**CDK5R1**

**Name:** cyclin-dependent kinase 5, regulatory subunit 1 (p35)

**Aliases:** CDK5 activator 1, CDK5P35, CDK5R, Cyclin-dependent kinase 5 activator 1, Cyclin-dependent kinase 5 regulatory subunit 1, MGC33831, Nck5a, NCK5A, p23, p25, p35, p35nck5a, Tau protein kinase II 23 kDa subunit, TPKII regulatory subunit

<http://www.ihop-net.org/UniPub/iHOP/mgi/94201.html>

**CHRNA1**

**Name:** cholinergic receptor, nicotinic, alpha 1 (muscle)

**Aliases:** Acetylcholine receptor subunit alpha, ACHRA, ACHRD, CHNRA, CHRNA, CMS2A, FCCMS, SCCMS

<http://www.ihop-net.org/UniPub/iHOP/mgi/87199.html>

**CHRNA9**

**Name:** cholinergic receptor, nicotinic, alpha 9

**Aliases:** HSA243342, MGC142109, MGC142135, NACHRA9, NACHR alpha 9, Neuronal acetylcholine receptor subunit alpha-9, Nicotinic acetylcholine receptor subunit alpha 9

<http://www.ihop-net.org/UniPub/iHOP/mgi/100643.html>

**CHRNB3**

**Name:** cholinergic receptor, nicotinic, beta 3

**Aliases:** Neuronal acetylcholine receptor subunit beta-3

<http://www.ihop-net.org/UniPub/iHOP/mgi/87207.html>

**CLIM1**

***See LDB2***

**COLQ**

**Name:** collagen-like tail subunit (single strand of homotrimer) of asymmetric acetylcholinesterase

**Aliases:** Acetylcholinesterase-associated collagen, Acetylcholinesterase collagenic tail peptide, AChE Q subunit, EAD, FLJ55041

<http://www.ihop-net.org/UniPub/iHOP/mgi/93720.html>

**COX6A1**

**Name:** cytochrome c oxidase subunit VIa polypeptide 1

**Aliases:** COX6A, COX6AL, Cytochrome c oxidase polypeptide VIa-liver, Cytochrome c oxidase subunit 6A1, mitochondrial, MGC104500

<http://www.ihop-net.org/UniPub/iHOP/mgi/87373.html>

**CREBBP**

**Name:** CREB binding protein

**Aliases:** CBP, CREB-binding protein, KAT3A, RSTS, RTS

<http://www.ihop-net.org/UniPub/iHOP/mgi/87420.html>

**CSF1**

**Name:** colony stimulating factor 1 (macrophage)

**Aliases:** CSF-1, Lanimostim, Macrophage colony-stimulating factor 1, MCSF, M-CSF, MGC31930

<http://www.ihop-net.org/UniPub/iHOP/mgi/87465.html>

**CSF3**

**Name:** colony stimulating factor 3 (granulocyte)

**Aliases:** GCSF, G-CSF, Granulocyte colony-stimulating factor, MGC45931, Pluripoietin

<http://www.ihop-net.org/UniPub/iHOP/mgi/87470.html>

**CSNK1A1**

**Name:** casein kinase 1, alpha 1

**Aliases:** Casein kinase I isoform alpha, CK1, CKI-alpha, HLCDGP1, PRO2975

<http://www.ihop-net.org/UniPub/iHOP/mgi/87479.html>

**CSNK1G3**

**Name:** casein kinase 1, gamma 3

**Aliases:** Casein kinase I isoform gamma-3, CKI-gamma 3

<http://www.ihop-net.org/UniPub/iHOP/mgi/87483.html>

**CSNK2A2**

**Name:** casein kinase 2, alpha prime polypeptide

**Aliases:** Casein kinase II subunit alpha', CK2A2, CK II, CSNK2A1, FLJ43934

<http://www.ihop-net.org/UniPub/iHOP/mgi/87485.html>

**CTBP1**

**Name:** C-terminal binding protein 1

**Aliases:** BARS, CTBP, CtBP1, C-terminal-binding protein 1, MGC104684

<http://www.ihop-net.org/UniPub/iHOP/mgi/87512.html>

**CTBP2**

**Name:** C-terminal binding protein 2

**Aliases:** CtBP2, C-terminal-binding protein 2, ribeye

<http://www.ihop-net.org/UniPub/iHOP/mgi/87513.html>

**DCC**

**Name:** deleted in colorectal carcinoma

**Aliases:** Colorectal cancer suppressor, CRC18, CRCR1, IGDCC1, Immunoglobulin superfamily DCC subclass member 1, Netrin receptor DCC, Tumor suppressor protein DCC

<http://www.ihop-net.org/UniPub/iHOP/mgi/87642.html>

**DLG1**

**Name:** discs, large homolog 1 (Drosophila)

**Aliases:** Disks large homolog 1, dJ1061C18.1.1, DKFZp761P0818, DKFZp781B0426, DLGH1, hdlg, hDlg, SAP97, SAP-97, Synapse-associated protein 97

<http://www.ihop-net.org/UniPub/iHOP/mgi/87730.html>

**DLG5**

**Name:** discs, large homolog 5 (Drosophila)

**Aliases:** Discs large protein P-dlg, Disks large homolog 5, KIAA0583, LP-DLG, PDLG, P-dlg, P-DLG5, Placenta and prostate DLG

<http://www.ihop-net.org/UniPub/iHOP/mgi/94487.html>

**DLGAP2**

**Name:** discs, large (Drosophila) homolog-associated protein 2

**Aliases:** DAP2, DAP-2, Disks large-associated protein 2, PSD-95/SAP90-binding protein 2, SAP90/PSD-95-associated protein 2, SAPAP2

<http://www.ihop-net.org/UniPub/iHOP/mgi/94484.html>

**DMD**

**Name:** dystrophin

**Aliases:** BMD, CMD3B, DXS142, DXS164, DXS206, DXS230, DXS239, DXS268, DXS269, DXS270, DXS272, Dystrophin

<http://www.ihop-net.org/UniPub/iHOP/mgi/87743.html>

**E2F1**

**Name:** E2F transcription factor 1

**Aliases:** E2F-1, PBR3, PRB-binding protein E2F-1, RBAP1, RBAP-1, RBBP3, RBBP-3, RBP3, Retinoblastoma-associated protein 1, Retinoblastoma-binding protein 3, Transcription factor E2F1

<http://www.ihop-net.org/UniPub/iHOP/mgi/87848.html>

**EGF**

**Name:** epidermal growth factor (beta-urogastrone)

**Aliases:** HOMG4, Pro-epidermal growth factor, URG

<http://www.ihop-net.org/UniPub/iHOP/mgi/87917.html>

**EGR**

***See EGR1 and EGR2***

**EGR1**

**Name:** early growth response 1

**Aliases:** AT225, Early growth response protein 1, EGR-1, G0S30, KROX-24, Nerve growth factor-induced protein A, NGFI-A, Protein Krox-24, TIS8, Transcription factor ETR103, Transcription factor Zif268, ZIF-268, Zinc finger protein 225, ZNF225

<http://www.ihop-net.org/UniPub/iHOP/mgi/87925.html>

**EGR2**

**Name:** early growth response 2

**Aliases:** AT591, CMT1D, CMT4E, DKFZp686J1957, Early growth response protein 2, EGR-2, FLJ14547, KROX20, Protein Krox-20

<http://www.ihop-net.org/UniPub/iHOP/mgi/87926.html>

**EGR3**

**Name:** early growth response 3

**Aliases:** Early growth response protein 3, EGR-3, MGC138484, PILOT, Zinc finger protein pilot

<http://www.ihop-net.org/UniPub/iHOP/mgi/87927.html>

**EIF4E2**

**Name:** eukaryotic translation initiation factor 4E family member 2

**Aliases:** 4EHP, 4E-LP, EIF4EL3, eIF4E-like protein 4E-LP, eIF4E type 2, eIF-4E type 2, Eukaryotic translation initiation factor 4E homologous protein, Eukaryotic translation initiation factor 4E-like 3, Eukaryotic translation initiation factor 4E type 2, IF4e, mRNA cap-binding protein 4EHP, mRNA cap-binding protein type 3

<http://www.ihop-net.org/UniPub/iHOP/mgi/94681.html>

**ELOVL2**

**Name:** elongation of very long chain fatty acids (FEN1/Elo2, SUR4/Elo3, yeast)-like 2

**Aliases:** Elongation of very long chain fatty acids protein 2, FLJ20334, Ssc2, SSC2

<http://www.ihop-net.org/UniPub/iHOP/mgi/100150.html>

**ERBB4**

**Name:** v-erb-a erythroblastic leukemia viral oncogene homolog 4 (avian)

**Aliases:** HER4, MGC138404, p180erbB4, Receptor tyrosine-protein kinase erbB-4, Tyrosine kinase-type cell surface receptor HER4

<http://www.ihop-net.org/UniPub/iHOP/mgi/88025.html>

**EVI1**

**Name:** ecotropic viral integration site 1

**Aliases:** AML1-EVI-1, Ecotropic virus integration site 1 protein homolog, EVI-1, MDS1-EVI1, MGC163392, PRDM3

<http://www.ihop-net.org/UniPub/iHOP/mgi/88076.html>

**FAIM2**

**Name:** Fas apoptotic inhibitory molecule 2

**Aliases:** KIAA0950, LFG, LIFEGUARD, NGP35, NMP35, Protein lifeguard, TMBIM2, Transmembrane BAX inhibitor motif-containing protein 2

<http://www.ihop-net.org/UniPub/iHOP/mgi/96499.html>

**FDFT1**

**Name:** farnesyl-diphosphate farnesyltransferase 1

**Aliases:** DGPT, ERG9, Farnesyl-diphosphate farnesyltransferase, FPP:FPP farnesyltransferase, SQS, Squalene synthetase, SS

<http://www.ihop-net.org/UniPub/iHOP/mgi/88171.html>

**FEZ1**

**Name:** fasciculation and elongation protein zeta 1 (zygin I)

**Aliases:** Fasciculation and elongation protein zeta-1, Zygin-1, Zygin I

<http://www.ihop-net.org/UniPub/iHOP/mgi/94824.html>

**FOXO1**

**Name:** forkhead box O1

**Aliases:** FKH1, FKHR, Forkhead box protein O1, Forkhead box protein O1A, Forkhead in rhabdomyosarcoma, FOXO1A

<http://www.ihop-net.org/UniPub/iHOP/mgi/88252.html>

**FOXO1A**

***See FOXO1***

**FRAT1**

**Name:** frequently rearranged in advanced T-cell lymphomas

**Aliases:** Frat-1, Frequently rearranged in advanced T-cell lymphomas, Proto-oncogene FRAT1

<http://www.ihop-net.org/UniPub/iHOP/mgi/95170.html>

**FTL**

**Name:** ferritin, light polypeptide

**Aliases:** Ferritin light chain, Ferritin L subunit, MGC71996

<http://www.ihop-net.org/UniPub/iHOP/mgi/88450.html>

**FYCO1**

**Name:** FYVE and coiled-coil domain containing 1

**Aliases:** DKFZp779K1152, FLJ13335, FYVE and coiled-coil domain-containing protein 1, MGC126517, MGC126519, RUFY3, ZFYVE7, Zinc finger FYVE domain-containing protein 7

<http://www.ihop-net.org/UniPub/iHOP/mgi/102619.html>

**FZD5**

**Name:** frizzled homolog 5 (Drosophila)

**Aliases:** C2orf31, chromosome 2 open reading frame 31, DKFZp434E2135, DKFZP434E2135, Frizzled-5, Fz-5, FzE5, hFz5, HFZ5, MGC129692

<http://www.ihop-net.org/UniPub/iHOP/mgi/93421.html>

**G-CSF**

***See CSF3***

**GABBR2**

**Name:** gamma-aminobutyric acid (GABA) B receptor, 2

**Aliases:** FLJ36928, GABABR2, GABA-B-R2, GABA-B receptor 2, Gamma-aminobutyric acid type B receptor subunit 2, Gb2, GPR51, GPRC3B, G-protein coupled receptor 51, HG20, HRIHFB2099

<http://www.ihop-net.org/UniPub/iHOP/mgi/94765.html>

**GABRA5**

**Name:** gamma-aminobutyric acid (GABA) A receptor, alpha 5

**Aliases:** GABA(A) receptor subunit alpha-5, Gamma-aminobutyric acid receptor subunit alpha-5

<http://www.ihop-net.org/UniPub/iHOP/mgi/88493.html>

**GABRQ**

**Name:** gamma-aminobutyric acid (GABA) receptor, theta

**Aliases:** GABA(A) receptor subunit theta, Gamma-aminobutyric acid receptor subunit theta, MGC129629, MGC129630, THETA

<http://www.ihop-net.org/UniPub/iHOP/mgi/100884.html>

**GLRA3**

**Name:** glycine receptor, alpha 3

**Aliases:** Glycine receptor subunit alpha-3

<http://www.ihop-net.org/UniPub/iHOP/mgi/93524.html>

**GLUR2**

***See GRIA***

**GLUR7**

***See GRM7***

**GNA14**

**Name:** guanine nucleotide binding protein (G protein), alpha 14

**Aliases:** G alpha-14, G-protein subunit alpha-14, Guanine nucleotide-binding protein subunit alpha-14

<http://www.ihop-net.org/UniPub/iHOP/mgi/94816.html>

**GNAS**

**Name:** GNAS complex locus

**Aliases:** Adenylate cyclase-stimulating G alpha protein, AHO, Alternative gene product encoded by XL-exon, C20orf45, dJ309F20.1.1, dJ806M20.3.3, Extra large alphas protein, GNAS1, GNASXL, GPSA, GSA, GSP, Guanine nucleotide-binding protein G(s) subunit alpha isoforms short, Guanine nucleotide-binding protein G(s) subunit alpha isoforms XLas, MGC33735, NESP, NESP55, Neuroendocrine secretory protein 55, PHP1A, PHP1B, POH, Protein ALEX, SCG6, XLalphas

<http://www.ihop-net.org/UniPub/iHOP/mgi/88704.html>

**GRIA**

**Name:** glutamate receptor, ionotropic, AMPA 2

**Aliases:** AMPA-selective glutamate receptor 2, GLUR2, GluR-2, GLURB, GluR-B, GluR-K2, Glutamate receptor 2, Glutamate receptor ionotropic, AMPA 2, HBGR2

<http://www.ihop-net.org/UniPub/iHOP/mgi/88814.html>

**GRIA2**

**Name:** glutamate receptor, ionotropic, AMPA 2

**Aliases:** AMPA-selective glutamate receptor 2, GLUR2, GluR-2, GLURB, GluR-B, GluR-K2, Glutamate receptor 2, Glutamate receptor ionotropic, AMPA 2, HBGR2

<http://www.ihop-net.org/UniPub/iHOP/mgi/88814.html>

**GRIK3**

**Name:** glutamate receptor, ionotropic, kainate 3

**Aliases:** EAA5, Excitatory amino acid receptor 5, GLR7, GluR7, GLUR7, GluR-7, GluR7a, Glutamate receptor, ionotropic kainate 3, Glutamate receptor 7

<http://www.ihop-net.org/UniPub/iHOP/mgi/88822.html>

**GRM5**

**Name:** glutamate receptor, metabotropic 5

**Aliases:** GPRC1E, Metabotropic glutamate receptor 5, mGlu5, mGluR5, MGLUR5

<http://www.ihop-net.org/UniPub/iHOP/mgi/88838.html>

**GRM7**

**Name:** glutamate receptor, metabotropic 7

**Aliases:** FLJ40498, GLUR7, GPRC1G, Metabotropic glutamate receptor 7, mGlu7, mGluR7, MGLUR7

<http://www.ihop-net.org/UniPub/iHOP/mgi/88840.html>

**GSTA3**

**Name:** glutathione S-transferase alpha 3

**Aliases:** Glutathione S-transferase A3, Glutathione S-transferase A3-3, GSTA3-3, GST class-alpha member 3, GTA3, MGC22232

<http://www.ihop-net.org/UniPub/iHOP/mgi/88861.html>

**GSTM4**

**Name:** glutathione S-transferase mu 4

**Aliases:** Glutathione S-transferase Mu 4, GST class-mu 4, GSTM4-4, GTM4, GTS-Mu2, MGC131945, MGC9247

<http://www.ihop-net.org/UniPub/iHOP/mgi/88869.html>

**HBEGF**

**Name:** heparin-binding EGF-like growth factor

**Aliases:** DTR, DTS, DTSF, HEGFL, Proheparin-binding EGF-like growth factor

<http://www.ihop-net.org/UniPub/iHOP/mgi/87819.html>

**HNF1**

***See NF1***

**HNF1A**

**Name:** HNF1 homeobox A

**Aliases:** Hepatocyte nuclear factor 1-alpha, HNF1, HNF-1A, LFB1, Liver-specific transcription factor LF-B1, MODY3, TCF1, TCF-1, Transcription factor 1

<http://www.ihop-net.org/UniPub/iHOP/mgi/92576.html>

**HNF1B**

**Name:** HNF1 homeobox B

**Aliases:** FJHN, Hepatocyte nuclear factor 1-beta, HNF-1B, HNF1beta, HNF-1-beta, HNF2, Homeoprotein LFB3, HPC11, LFB3, LF-B3, MODY5, TCF2, TCF-2, Transcription factor 2, Variant hepatic nuclear factor 1, vHNF1, VHNF1

<http://www.ihop-net.org/UniPub/iHOP/mgi/92577.html>

**HOMER3**

**Name:** homer homolog 3 (Drosophila)

**Aliases:** Homer-3, HOMER-3, Homer protein homolog 3, VESL3

<http://www.ihop-net.org/UniPub/iHOP/mgi/94668.html>

**HTR2A**

**Name:** 5-hydroxytryptamine (serotonin) receptor 2A

**Aliases:** 5-HT-2, 5-HT2A, 5-HT-2A, 5-hydroxytryptamine receptor 2A, HTR2, Serotonin receptor 2A

<http://www.ihop-net.org/UniPub/iHOP/mgi/89255.html>

**ICA1**

**Name:** islet cell autoantigen 1, 69kDa

**Aliases:** 69 kDa islet cell autoantigen, ICA69, ICAp69, Islet cell autoantigen 1, Islet cell autoantigen p69, p69

<http://www.ihop-net.org/UniPub/iHOP/mgi/89277.html>

**IL-3**

***See IL3***

**IL-6**

***See IL6***

**IL1-a**

***See IL1A***

**IL1A**

**Name:** interleukin 1, alpha

**Aliases:** Hematopoietin-1, IL1, IL-1A, IL1-ALPHA, IL-1 alpha, IL1F1, Interleukin-1 alpha

<http://www.ihop-net.org/UniPub/iHOP/gs/89437.html>

**IL3**

**Name:** interleukin 3 (colony-stimulating factor, multiple)

**Aliases:** Hematopoietic growth factor, IL-3, Interleukin-3, Mast cell growth factor, MCGF, MGC79398, MGC79399, MULTI-CSF, Multipotential colony-stimulating factor, P-cell-stimulating factor

<http://www.ihop-net.org/UniPub/iHOP/mgi/89446.html>

**IL6**

**Name:** interleukin 6 (interferon, beta 2)

**Aliases:** B-cell stimulatory factor 2, BSF2, BSF-2, CDF, CTL differentiation factor, HGF, HSF, Hybridoma growth factor, IFNB2, IL-6, Interferon beta-2, Interleukin-6

<http://www.ihop-net.org/UniPub/iHOP/mgi/89452.html>

**INSR**

**Name:** insulin receptor

**Aliases:** CD220, HHF5, Insulin receptor, IR

<http://www.ihop-net.org/UniPub/iHOP/mgi/89524.html>

**ITGB1**

**Name:** integrin, beta 1 (fibronectin receptor, beta polypeptide, antigen CD29 includes MDF2, MSK12)

**Aliases:** CD29, Fibronectin receptor subunit beta, FNRB, GPIIA, Integrin beta-1, Integrin VLA-4 subunit beta, MDF2, MSK12, VLAB, VLA-BETA

<http://www.ihop-net.org/UniPub/iHOP/mgi/89566.html>

**ITPR1**

**Name:** inositol 1,4,5-triphosphate receptor, type 1

**Aliases:** Inositol 1,4,5-trisphosphate receptor type 1, Insp3r1, InsP3R1, INSP3R1, IP3R, IP3R1, IP3 receptor isoform 1, SCA15, SCA16, Type 1 inositol 1,4,5-trisphosphate receptor, Type 1 InsP3 receptor

<http://www.ihop-net.org/UniPub/iHOP/mgi/89585.html>

**ITPR2**

**Name:** inositol 1,4,5-triphosphate receptor, type 2

**Aliases:** Inositol 1,4,5-trisphosphate receptor type 2, InsP3R2, IP3R2, IP3 receptor isoform 2, Type 2 inositol 1,4,5-trisphosphate receptor, Type 2 InsP3 receptor

<http://www.ihop-net.org/UniPub/iHOP/mgi/89586.html>

**KIAA0528**

**Name:** KIAA0528

**Aliases:** Uncharacterized protein KIAA0528

<http://www.ihop-net.org/UniPub/iHOP/mgi/95011.html>

**KROX24**

***See ERG1***

**LDB2**

**Name:** LIM domain binding 2

**Aliases:** Carboxyl-terminal LIM domain-binding protein 1, CLIM1, CLIM-1, LDB1, LIM domain-binding factor CLIM1, LIM domain-binding protein 2

<http://www.ihop-net.org/UniPub/iHOP/mgi/94360.html>

**LDHA**

**Name:** lactate dehydrogenase A

**Aliases:** Cell proliferation-inducing gene 19 protein, LDH1, LDH-A, LDH-M, LDH muscle subunit, L-lactate dehydrogenase A chain, PIG19, Renal carcinoma antigen NY-REN-59

<http://www.ihop-net.org/UniPub/iHOP/mgi/89804.html>

**LF-B1**

**See HNF1A**

**LHCGR**

**Name:** luteinizing hormone/choriogonadotropin receptor

**Aliases:** FLJ41504, LCGR, LGR2, LH/CGR, LH/CG-R, LHR, LHRHR, LSH-R, Luteinizing hormone receptor, Lutropin-choriogonadotropic hormone receptor

<http://www.ihop-net.org/UniPub/iHOP/mgi/89834.html>

**LMO4**

**Name:** LIM domain only 4

**Aliases:** Breast tumor autoantigen, LIM domain only protein 4, LIM domain transcription factor LMO4, LMO-4

<http://www.ihop-net.org/UniPub/iHOP/mgi/93937.html>

**LTF**

**Name:** lactotransferrin

**Aliases:** GIG12, HLF2, Lactoferrin, Lactotransferrin, LF, Talalactoferrin

<http://www.ihop-net.org/UniPub/iHOP/mgi/89908.html>

**LZTS1**

**Name:** leucine zipper, putative tumor suppressor 1

**Aliases:** F37, F37/esophageal cancer-related gene-coding leucine-zipper motif, Fez1, FEZ1

<http://www.ihop-net.org/UniPub/iHOP/mgi/96186.html>

**MAZ**

**Name:** MYC-associated zinc finger protein (purine-binding transcription factor)

**Aliases:** MAZI, Myc-associated zinc finger protein, PUR1, Pur-1, Purine-binding transcription factor, SAF-1, SAF-2, ZF87, Zif87, ZIF87, ZNF801

<http://www.ihop-net.org/UniPub/iHOP/mgi/89994.html>

**MAPK1**

**Name:** mitogen-activated protein kinase 1

**Aliases:** ERK, ERK2, ERK-2, ERT1, Extracellular signal-regulated kinase 2, MAPK2, MAPK 2, MAP kinase 2, Mitogen-activated protein kinase 1, Mitogen-activated protein kinase 2, p38, p40, p41, p41mapk, P42MAPK, p42-MAPK, PRKM1, PRKM2

<http://www.ihop-net.org/UniPub/iHOP/mgi/91323.html>

**MDFIC**

**Name:** MyoD family inhibitor domain containing

**Aliases:** HIC, I-mfa domain-containing protein, MyoD family inhibitor domain-containing protein

<http://www.ihop-net.org/UniPub/iHOP/mgi/98890.html>

**MCTP1**

**Name:** multiple C2 domains, transmembrane 1

**Aliases:** FLJ22344, Multiple C2 and transmembrane domain-containing protein 1

<http://www.ihop-net.org/UniPub/iHOP/mgi/102830.html>

**miR-9**

***See MIRN9-1***

**miR-128**

***See MIR128-1***

**miR-124a**

***See MIR124-1***

**miR-125a**

***See MIR125A***

**miR-125b**

<http://journals.lww.com/neuroreport/Abstract/2007/02120/Micro_RNA_speciation_in_fetal,_adult_and.20.aspx>

**miR-132**

***See MIR132***

**miR-219**

***See MIRN219-1***

**MIR124-1**

**Name:** microRNA 124-1

**Aliases:** hsa-mir-124-1, hsa-mir-124a-1, MIR124A, MIR124A1, MIRN124-1, MIRN124A1

<http://www.ihop-net.org/UniPub/iHOP/mgi/118871.html>

**MIR125A**

**Name:** microRNA 125a

**Aliases:** hsa-mir-125a, MIRN125A, miRNA125A

<http://www.ihop-net.org/UniPub/iHOP/mgi/118874.html>

**MIR128-1**

**Name:** microRNA 128-1

**Aliases:** hsa-mir-128-1, hsa-mir-128a, MIR128A, MIRN128-1, MIRN128A

<http://www.ihop-net.org/UniPub/iHOP/mgi/118879.html>

**MIR132**

**Name:** microRNA 132

**Aliases:** hsa-mir-132, MIRN132, miRNA132

<http://www.ihop-net.org/UniPub/iHOP/mgi/118885.html>

**MIRN9-1**

**Name:** microRNA 9-1

**Aliases:** hsa-mir-9-1, MIRN9-1, miRNA9-1

<http://www.ihop-net.org/UniPub/iHOP/mgi/119010.html>

**MIRN219-1**

**Name:** microRNA 219-1

**Aliases:** hsa-mir-219-1, mir-219, MIRN219-1, MRI219-1

<http://www.ihop-net.org/UniPub/iHOP/mgi/118966.html>

**MT1H**

**Name:** metallothionein 1H

**Aliases:** Metallothionein-0, Metallothionein-1H, Metallothionein-IH, MGC70702, MT-0, MT1, MT-1H, MT-IH

<http://www.ihop-net.org/UniPub/iHOP/mgi/90312.html>

**MT1F**

**Name:** metallothionein 1F

**Aliases:** Metallothionein-1F, Metallothionein-IF, MGC32732, MT1, MT-1F, MT-IF, PRO0376

<http://www.ihop-net.org/UniPub/iHOP/mgi/90310.html>

**MT1L**

**Name:** metallothionein 1L (gene/pseudogene)

**Aliases:** Metallothionein-1L, Metallothionein-IL, MT1, MT-1L, MT1R, MTF, MT-IL

<http://www.ihop-net.org/UniPub/iHOP/mgi/90316.html>

**MTF1**

**Name:** metal-regulatory transcription factor 1

**Aliases:** Metal regulatory transcription factor 1, MGC23036, MRE-binding transcription factor, MTF-1, Transcription factor MTF-1, ZRF

<http://www.ihop-net.org/UniPub/iHOP/mgi/90330.html>

**NEFL**

**Name:** neurofilament, light polypeptide

**Aliases:** 68 kDa neurofilament protein, CMT1F, CMT2E, FLJ53642, Neurofilament light polypeptide, Neurofilament triplet L protein, NF68, NFL, NF-L

<http://www.ihop-net.org/UniPub/iHOP/mgi/90533.html>

**NEFM**

**Name:** neurofilament, medium polypeptide

**Aliases:** 160 kDa neurofilament protein, NEF3, Neurofilament 3, Neurofilament medium polypeptide, Neurofilament triplet M protein, NFM, NF-M

<http://www.ihop-net.org/UniPub/iHOP/mgi/90529.html>

**NF-M**

***See NEFM***

**NF1**

**Name:** neurofibromin 1

**Aliases:** DKFZp686J1293, FLJ21220, Neurofibromatosis-related protein NF-1, Neurofibromin, NFNS, VRNF, WSS

<http://www.ihop-net.org/UniPub/iHOP/mgi/90547.html>

**NFL**

***See NEFL***

**NDUFV2**

**Name:** NADH dehydrogenase (ubiquinone) flavoprotein 2, 24kDa

**Aliases:** NADH-ubiquinone oxidoreductase 24 kDa subunit

<http://www.ihop-net.org/UniPub/iHOP/mgi/90518.html>

**NFATC1**

**Name:** nuclear factor of activated T-cells, cytoplasmic, calcineurin-dependent 1

**Aliases:** MGC138448, NFAT2, NFATc, NFATC, NF-ATc, NF-ATC, NF-ATc1, NFAT transcription complex cytosolic component, Nuclear factor of activated T-cells, cytoplasmic 1

<http://www.ihop-net.org/UniPub/iHOP/mgi/90556.html>

**NRG1**

**Name:** neuregulin 1

**Aliases:** ARIA, GGF, GGF2, HGL, HRG, HRG1, HRGA, NDF, Neuregulin-1, sensory and motor neuron-derived factor isoform, Pro-neuregulin-1, membrane-bound isoform, Pro-NRG1, SMDF

<http://www.ihop-net.org/UniPub/iHOP/mgi/88998.html>

**NRXN1**

**Name:** neurexin 1

**Aliases:** DKFZp313P2036, FLJ35941, Hs.22998, KIAA0578, Neurexin-1-alpha, Neurexin-1-beta, Neurexin I-alpha, Neurexin I-beta

<http://www.ihop-net.org/UniPub/iHOP/mgi/94604.html>

**NUFIP1**

**Name:** nuclear fragile X mental retardation protein interacting protein 1

**Aliases:** bA540M5.1, Nuclear FMRP-interacting protein 1, Nuclear fragile X mental retardation-interacting protein 1, NUFIP

<http://www.ihop-net.org/UniPub/iHOP/mgi/97690.html>

**NXN**

**Name:** nucleoredoxin

**Aliases:** FLJ12614, NRX, Nucleoredoxin, TRG-4

<http://www.ihop-net.org/UniPub/iHOP/mgi/102077.html>

**PAX6**

**Name:** paired box 6

**Aliases:** AN, AN2, Aniridia type II protein, D11S812E, MGC17209, MGDA, Oculorhombin, Paired box protein Pax-6, WAGR

<http://www.ihop-net.org/UniPub/iHOP/mgi/90834.html>

**PBR**

***See TSPO***

**PDE3A**

**Name:** phosphodiesterase 3A, cGMP-inhibited

**Aliases:** CGI-PDE, CGI-PDE A, cGMP-inhibited 3',5'-cyclic phosphodiesterase A, Cyclic GMP-inhibited phosphodiesterase A

<http://www.ihop-net.org/UniPub/iHOP/mgi/90889.html>

**PDH**

***See PDP1***

**PDHA1**

**Name:** pyruvate dehydrogenase (lipoamide) alpha 1

**Aliases:** PDHA, PDHCE1A, PDHE1-A type I, PHE1A, Pyruvate dehydrogenase E1 component subunit alpha, somatic form, mitochondrial

<http://www.ihop-net.org/UniPub/iHOP/mgi/90910.html>

**PDP1**

**Name:** pyruvate dehyrogenase phosphatase catalytic subunit 1

**Aliases:** FLJ32517, FLJ56179, MGC119646, PDH, PDP, PDP 1, PDPC, PDPC 1, PPM2C, Protein phosphatase 2C, Pyruvate dehydrogenase phosphatase, catalytic subunit 1

<http://www.ihop-net.org/UniPub/iHOP/mgi/100009.html>

**PHKA2**

**Name:** phosphorylase kinase, alpha 2 (liver)

**Aliases:** GSD9A, MGC133071, PHK, PHKLA, Phosphorylase b kinase regulatory subunit alpha, liver isoform, Phosphorylase kinase alpha L subunit, PYK, PYKL, XLG, XLG2

<http://www.ihop-net.org/UniPub/iHOP/mgi/90999.html>

**PIAS4**

**Name:** protein inhibitor of activated STAT, 4

**Aliases:** E3 SUMO-protein ligase PIAS4, FLJ12419, MGC35296, Piasg, PIASG, PIAS-gamma, PIASy, PIASY, Protein inhibitor of activated STAT protein 4, Protein inhibitor of activated STAT protein gamma, ZMIZ6

<http://www.ihop-net.org/UniPub/iHOP/mgi/99522.html>

**PIK3C3**

**Name:** phosphoinositide-3-kinase, class 3

**Aliases:** MGC61518, Phosphatidylinositol 3-kinase catalytic subunit type 3, Phosphatidylinositol 3-kinase p100 subunit, Phosphoinositide-3-kinase class 3, PI3-kinase type 3, PI3K type 3, PtdIns-3-kinase type 3, Vps34, VPS34

<http://www.ihop-net.org/UniPub/iHOP/mgi/91032.html>

**PIK3R1**

**Name:** phosphoinositide-3-kinase, regulatory subunit 1 (alpha)

**Aliases:** GRB1, p85, p85-ALPHA, Phosphatidylinositol 3-kinase regulatory subunit alpha, PI3K, PI3-kinase p85 subunit alpha, PtdIns-3-kinase p85-alpha

**PIK3R4**

**Name:** phosphoinositide-3-kinase, regulatory subunit 4

**Aliases:** MGC102700, p150, Phosphoinositide 3-kinase adaptor protein, Phosphoinositide 3-kinase regulatory subunit 4, PI3-kinase p150 subunit, PI3-kinase regulatory subunit 4, VPS15

<http://www.ihop-net.org/UniPub/iHOP/mgi/98947.html>

**PI4KB**

**Name:** phosphatidylinositol 4-kinase, catalytic, beta

**Aliases:** NPIK, Phosphatidylinositol 4-kinase beta, pi4K92, PI4K92, PI4Kbeta, PI4KBETA, PI4K-beta, PI4K-BETA, PI4KIIIBETA, PIK4CB, PtdIns 4-kinase beta

<http://www.ihop-net.org/UniPub/iHOP/mgi/91041.html>

**PIP5K1A**

**Name:** phosphatidylinositol-4-phosphate 5-kinase, type I, alpha

**Aliases:** 68 kDa type I phosphatidylinositol-4-phosphate 5-kinase alpha, Phosphatidylinositol-4-phosphate 5-kinase type-1 alpha, Phosphatidylinositol-4-phosphate 5-kinase type I alpha, PIP5KIalpha, PtdIns(4)P-5-kinase alpha

<http://www.ihop-net.org/UniPub/iHOP/mgi/93807.html>

**PIP5K1C**

**Name:** phosphatidylinositol-4-phosphate 5-kinase, type I, gamma

**Aliases:** KIAA0589, LCCS3, Phosphatidylinositol-4-phosphate 5-kinase type-1 gamma, Phosphatidylinositol-4-phosphate 5-kinase type I gamma, PIP5Kgamma, PIP5K-GAMMA, PIP5KIgamma, PtdIns(4)P-5-kinase gamma, PtdInsPKIgamma

<http://www.ihop-net.org/UniPub/iHOP/mgi/96812.html>

**PIP4K2C**

**Name:** phosphatidylinositol-5-phosphate 4-kinase, type II, gamma

**Aliases:** FLJ22055, Phosphatidylinositol-5-phosphate 4-kinase type-2 gamma, Phosphatidylinositol-5-phosphate 4-kinase type II gamma, PI(5)P 4-kinase type II gamma, PIP4KII-gamma, PIP5K2C

<http://www.ihop-net.org/UniPub/iHOP/mgi/102883.html>

**PLCB1**

**Name:** phospholipase C, beta 1 (phosphoinositide-specific)

**Aliases:** 1-phosphatidylinositol-4,5-bisphosphate phosphodiesterase beta-1, FLJ45792, KIAA0581, Phosphoinositide phospholipase C, Phospholipase C-beta-1, PI-PLC, PLC154, PLC-154, PLC-beta-1, PLC-I

<http://www.ihop-net.org/UniPub/iHOP/mgi/96682.html>

**PLCE1**

**Name:** phospholipase C, epsilon 1

**Aliases:** 1-phosphatidylinositol-4,5-bisphosphate phosphodiesterase epsilon-1, FLJ23659, KIAA1516, MGC167842, NPHS3, Pancreas-enriched phospholipase C, Phosphoinositide-specific phospholipase C epsilon-1, Phospholipase C-epsilon-1, PLCE, PLC-epsilon-1, PPLC

<http://www.ihop-net.org/UniPub/iHOP/mgi/99268.html>

**PP2A**

***See PPP2R4***

***PP2B***

***See PPP3CA and PPP3R1***

**PPARD**

**Name:** peroxisome proliferator-activated receptor delta

**Aliases:** FAAR, MGC3931, NR1C2, NUC1, NUCI, NUCII, Nuclear hormone receptor 1, Nuclear receptor subfamily 1 group C member 2, Peroxisome proliferator-activated receptor delta, PPARB, PPAR-beta, PPAR-delta

<http://www.ihop-net.org/UniPub/iHOP/mgi/91204.html>

**PPIA**

**Name:** peptidylprolyl isomerase A (cyclophilin A)

**Aliases:** Cyclophilin A, Cyclosporin A-binding protein, CYPA, CYPH, MGC117158, MGC12404, MGC23397, Peptidyl-prolyl cis-trans isomerase A, PPIase A, Rotamase A

<http://www.ihop-net.org/UniPub/iHOP/mgi/91215.html>

**PPP1CC**

**Name:** protein phosphatase 1, catalytic subunit, gamma isoform

**Aliases:** PP-1G, Protein phosphatase 1C catalytic subunit, Serine/threonine-protein phosphatase PP1-gamma catalytic subunit

<http://www.ihop-net.org/UniPub/iHOP/mgi/91236.html>

**PPP2B**

***See PPP3CA***

**PPP2CA**

**Name:** protein phosphatase 2 (formerly 2A), catalytic subunit, alpha isoform

**Aliases:** PP2A-alpha, PP2Ac, PP2CA, Replication protein C, RP-C, Serine/threonine-protein phosphatase 2A catalytic subunit alpha isoform

<http://www.ihop-net.org/UniPub/iHOP/mgi/91248.html>

**PPP2R2B**

**Name:** protein phosphatase 2 (formerly 2A), regulatory subunit B, beta isoform

**Aliases:** B55-BETA, FLJ95686, MGC24888, PP2A, subunit B, B55-beta isoform, PP2A, subunit B, B-beta isoform, PP2A, subunit B, PR55-beta isoform, PP2A, subunit B, R2-beta isoform, PP2A-B55BETA, PP2AB-BETA, PP2A-PR55B, PP2APR55-BETA, PR2AB55-BETA, PR2AB-BETA, PR2APR55-BETA, PR52B, PR55-BETA, SCA12, Serine/threonine-protein phosphatase 2A 55 kDa regulatory subunit B beta isoform

<http://www.ihop-net.org/UniPub/iHOP/mgi/91254.html>

**PPP2R4**

**Name:** protein phosphatase 2A activator, regulatory subunit 4

**Aliases:** MGC2184, Phosphotyrosyl phosphatase activator, PP2A, PP2A, subunit B', PR53 isoform, PR53, PTPA, Serine/threonine-protein phosphatase 2A regulatory subunit B'

<http://www.ihop-net.org/UniPub/iHOP/mgi/91257.html>

**PPP3CA**

**Name:** protein phosphatase 3 (formerly 2B), catalytic subunit, alpha isoform

**Aliases:** Calmodulin-dependent calcineurin A subunit alpha isoform, CALN, CALNA, CALNA1, CAM-PRP catalytic subunit, CCN1, CNA, CNA1, PPP2B, Serine/threonine-protein phosphatase 2B catalytic subunit alpha isoform

<http://www.ihop-net.org/UniPub/iHOP/mgi/91263.html>

**PPP3R1**

**Name:** protein phosphatase 3 (formerly 2B), regulatory subunit B, alpha isoform

**Aliases:** Calcineurin subunit B type 1, CALNB1, CNA2, CNB, CNB1, Protein phosphatase 2B regulatory subunit 1, Protein phosphatase 3 regulatory subunit B alpha isoform 1

<http://www.ihop-net.org/UniPub/iHOP/mgi/91267.html>

**PPT1**

**Name:** palmitoyl-protein thioesterase 1

**Aliases:** CLN1, INCL, Palmitoyl-protein hydrolase 1, Palmitoyl-protein thioesterase 1, PPT, PPT-1

<http://www.ihop-net.org/UniPub/iHOP/mgi/91271.html>

**PRKAR2A**

**Name:** protein kinase, cAMP-dependent, regulatory, type II, alpha

**Aliases:** cAMP-dependent protein kinase type II-alpha regulatory subunit, MGC3606, PKR2, PRKAR2

<http://www.ihop-net.org/UniPub/iHOP/mgi/91305.html>

**PRKAR2B**

**Name:** protein kinase, cAMP-dependent, regulatory, type II, beta

**Aliases:** cAMP-dependent protein kinase type II-beta regulatory subunit, PRKAR2, RII-BETA

<http://www.ihop-net.org/UniPub/iHOP/mgi/91306.html>

**PRKCB1**

**Name:** protein kinase C, beta

**Aliases:** MGC41878, PKCB, PKC-B, PKC-beta, PRKCB1, PRKCB2, Protein kinase C beta type

<http://www.ihop-net.org/UniPub/iHOP/mgi/91308.html>

**PRKCI**

**Name:** protein kinase C, iota

**Aliases:** aPKC-lambda/iota, Atypical protein kinase C-lambda/iota, DXS1179E, MGC26534, nPKC-iota, PKCI, PRKC-lambda/iota, Protein kinase C iota type

<http://www.ihop-net.org/UniPub/iHOP/mgi/91313.html>

**PSD3**

**Name:** pleckstrin and Sec7 domain containing 3

**Aliases:** DKFZp761K1423, EFA6R, Exchange factor for ADP-ribosylation factor guanine nucleotide factor 6, HCA67, Hepatocellular carcinoma-associated antigen 67, KIAA0942, PH and SEC7 domain-containing protein 3, Pleckstrin homology and SEC7 domain-containing protein 3

<http://www.ihop-net.org/UniPub/iHOP/mgi/96786.html>

**PTAFR**

**Name:** platelet-activating factor receptor

**Aliases:** PAFR, PAF-R, Platelet-activating factor receptor

<http://www.ihop-net.org/UniPub/iHOP/mgi/91447.html>

**PTEN**

**Name:** phosphatase and tensin homolog

**Aliases:** 10q23del, BZS, MGC11227, MHAM, MMAC1, Mutated in multiple advanced cancers 1, Phosphatase and tensin homolog, Phosphatidylinositol-3,4,5-trisphosphate 3-phosphatase and dual-specificity protein phosphatase PTEN, PTEN1, TEP1

<http://www.ihop-net.org/UniPub/iHOP/mgi/91451.html>

**QKI**

**Name:** quaking homolog, KH domain RNA binding (mouse)

**Aliases:** DKFZp586I0923, HKQ, Hqk, HqkI, Protein quaking, QK, QK1, QK3

<http://www.ihop-net.org/UniPub/iHOP/mgi/94659.html>

**RAB3B**

**Name:** RAB3B, member RAS oncogene family

**Aliases:** Ras-related protein Rab-3B

<http://www.ihop-net.org/UniPub/iHOP/mgi/91582.html>

**RBX1**

**Name:** ring-box 1

**Aliases:** BA554C12.1, MGC13357, MGC1481, Protein ZYP, Rbx1, Regulator of cullins 1, RING-box protein 1, RING finger protein 75, RNF75, ROC1

<http://www.ihop-net.org/UniPub/iHOP/mgi/95130.html>

**RHEB**

**Name:** Ras homolog enriched in brain

**Aliases:** GTP-binding protein Rheb, MGC111559, RHEB2

<http://www.ihop-net.org/UniPub/iHOP/mgi/91720.html>

**RHOQ**

**Name:** ras homolog gene family, member Q

**Aliases:** ARHQ, RASL7A, Ras-like protein family member 7A, Ras-related GTP-binding protein TC10, Rho-related GTP-binding protein RhoQ, TC10, TC10A

<http://www.ihop-net.org/UniPub/iHOP/mgi/96844.html>

**RIMS2**

**Name:** regulating synaptic membrane exocytosis 2

**Aliases:** DKFZp781A0653, KIAA0751, OBOE, Rab3-interacting molecule 2, RAB3IP3, Regulating synaptic membrane exocytosis protein 2, RIM2, RIM 2

<http://www.ihop-net.org/UniPub/iHOP/mgi/94878.html>

**RPS6KB2**

**Name:** ribosomal protein S6 kinase, 70kDa, polypeptide 2

**Aliases:** 70 kDa ribosomal protein S6 kinase 2, KLS, p70(S6K)-beta, p70-beta, P70-beta, P70-BETA, P70-beta-1, P70-beta-2, p70 ribosomal S6 kinase beta, p70S6Kb, p70-S6KB, p70 S6Kbeta, p70 S6 kinase beta, Ribosomal protein S6 kinase beta-2, S6K2, S6K-beta, S6K-beta2, S6K-beta 2, S6 kinase-related kinase, Serine/threonine-protein kinase 14 beta, SRK, STK14B

<http://www.ihop-net.org/UniPub/iHOP/mgi/91894.html>

**SAP97**

***See DLG1***

**SERTAD2**

**Name:** SERTA domain containing 2

**Aliases:** KIAA0127, MGC126688, MGC126690, Sei-2, SERTA domain-containing protein 2, Transcriptional regulator interacting with the PHD-bromodomain 2, TRIP-Br2

<http://www.ihop-net.org/UniPub/iHOP/mgi/94964.html>

**SHANK2**

**Name:** SH3 and multiple ankyrin repeat domains 2

**Aliases:** Cortactin-binding protein 1, CortBP1, CORTBP1, CTTNBP1, KIAA1022, ProSAP1, SH3 and multiple ankyrin repeat domains protein 2, SHANK, Shank2, SPANK-3

<http://www.ihop-net.org/UniPub/iHOP/mgi/96437.html>

**SKIP**

**Name:** inositol polyphosphate-5-phosphatase K

**Aliases:** Inositol polyphosphate 5-phosphatase K, PPS, Skeletal muscle and kidney-enriched inositol phosphatase, SKIP

<http://www.ihop-net.org/UniPub/iHOP/mgi/99618.html>

**SLC25A6**

**Name:** solute carrier family 25 (mitochondrial carrier; adenine nucleotide translocator), member 6

**Aliases:** AAC3, Adenine nucleotide translocator 2, ADP,ATP carrier protein, isoform T2, ADP,ATP carrier protein 3, ADP/ATP translocase 3, ANT3, ANT 3, ANT3Y, CDABP0051, MGC17525, Solute carrier family 25 member 6

<http://www.ihop-net.org/UniPub/iHOP/mgi/86422.html>

**SLC8A2**

**Name:** solute carrier family 8 (sodium/calcium exchanger), member 2

**Aliases:** KIAA1087, Na(+)/Ca(2+)-exchange protein 2, NCX2, Sodium/calcium exchanger 2

<http://www.ihop-net.org/UniPub/iHOP/mgi/92221.html>

**SMAD3**

**Name:** SMAD family member 3

**Aliases:** DKFZp586N0721, DKFZp686J10186, hMAD-3, hSMAD3, HSPC193, HsT17436, JV15-2, Mad3, MADH3, MGC60396, Mothers against decapentaplegic homolog 3, Mothers against DPP homolog 3, SMAD 3

<http://www.ihop-net.org/UniPub/iHOP/mgi/89937.html>

**SNPH**

**Name:** syntaphilin

**Aliases:** bA314N13.5, KIAA0374, MGC46096, Syntaphilin

<http://www.ihop-net.org/UniPub/iHOP/mgi/94927.html>

**SNRK**

**Name:** SNF related kinase

**Aliases:** DKFZp779A1866, FLJ20224, HSNFRK, KIAA0096, SNF1-related kinase, SNF-related serine/threonine-protein kinase, SNFRK

<http://www.ihop-net.org/UniPub/iHOP/mgi/100117.html>

**SOD1**

**Name:** superoxide dismutase 1, soluble

**Aliases:** ALS, ALS1, homodimer, IPOA, SOD

<http://www.ihop-net.org/UniPub/iHOP/mgi/92317.html>

**SOX2**

**Name:** SRY (sex determining region Y)-box 2

**Aliases:** ANOP3, MCOPS3, MGC2413, Transcription factor SOX-2

<http://www.ihop-net.org/UniPub/iHOP/mgi/92327.html>

**SNRPA**

**Name:**  small nuclear ribonucleoprotein polypeptide A

**Aliases:** U1A, U1-A, U1A protein, U1 small nuclear ribonucleoprotein A, U1 snRNP protein A

<http://www.ihop-net.org/UniPub/iHOP/mgi/92297.html>

**SSPN**

**Name:** sarcospan (Kras oncogene-associated gene)

**Aliases:** DAGA5, Kirsten-ras-associated protein, KRAG, K-ras oncogene-associated protein, nanospan, NSPN, Sarcospan, SPN1, SPN2

<http://www.ihop-net.org/UniPub/iHOP/mgi/93576.html>

**SV2B**

**Name:** synaptic vesicle glycoprotein 2B

**Aliases:** HsT19680, KIAA0735, Synaptic vesicle glycoprotein 2B

<http://www.ihop-net.org/UniPub/iHOP/mgi/95057.html>

**SYK**

**Name:** spleen tyrosine kinase

**Aliases:** DKFZp313N1010, FLJ25043, FLJ37489, Spleen tyrosine kinase, Tyrosine-protein kinase SYK

<http://www.ihop-net.org/UniPub/iHOP/mgi/92504.html>

**SYNC1**

**Name:** syncoilin, intermediate filament protein

**Aliases:** MGC149625, MGC149626, SYNC1, Syncoilin, SYNCOILIN, Syncoilin-1, Syncoilin intermediate filament 1

<http://www.ihop-net.org/UniPub/iHOP/mgi/103522.html>

**TBL1X**

**Name:** transducin (beta)-like 1X-linked

**Aliases:** EBI, F-box-like/WD repeat-containing protein TBL1X, SMAP55, TBL1, Transducin-beta-like protein 1, X-linked, Transducin beta-like protein 1X

<http://www.ihop-net.org/UniPub/iHOP/mgi/92557.html>

**TBXA2R**

**Name:** thromboxane A2 receptor

**Aliases:** Prostanoid TP receptor, Thromboxane A2 receptor, TXA2-R

<http://www.ihop-net.org/UniPub/iHOP/mgi/92564.html>

**TCF1**

***See HNF1A***

**TCF2**

***See HNF1B***

**TCF3**

**Name:** transcription factor 3 (E2A immunoglobulin enhancer binding factors E12/E47)

**Aliases:** bHLHb21, E2A, Immunoglobulin enhancer-binding factor E12/E47, Immunoglobulin transcription factor 1, ITF1, Kappa-E2-binding factor, MGC129647, MGC129648, TCF-3, Transcription factor 3, Transcription factor E2-alpha, Transcription factor ITF-1, VDIR

<http://www.ihop-net.org/UniPub/iHOP/mgi/92578.html>

**TCF7L1**

**Name:** transcription factor 7-like 1 (T-cell specific, HMG-box)

**Aliases:** HMG box transcription factor 3, TCF3, TCF-3, Transcription factor 7-like 1

<http://www.ihop-net.org/UniPub/iHOP/mgi/103663.html>

**TCF7L2**

**Name:** transcription factor 7-like 2 (T-cell specific, HMG-box)

**Aliases:** HMG box transcription factor 4, hTCF-4, T-cell-specific transcription factor 4, TCF4, TCF-4, Transcription factor 7-like 2

<http://www.ihop-net.org/UniPub/iHOP/mgi/92582.html>

**TLE3**

**Name:** transducin-like enhancer of split 3 (E(sp1) homolog, Drosophila)

**Aliases:** ESG, ESG3, FLJ39460, GRG3, HsT18976, KIAA1547, Transducin-like enhancer protein 3

<http://www.ihop-net.org/UniPub/iHOP/mgi/92732.html>

**TNF**

**Name:** tumor necrosis factor (TNF superfamily, member 2)

**Aliases:** Cachectin, DIF, TNFA, TNF-a, TNF-alpha, TNFSF2, Tumor necrosis factor, Tumor necrosis factor ligand superfamily member 2

<http://www.ihop-net.org/UniPub/iHOP/mgi/92766.html>

**TNF-a**

***See TNF***

**TNNC2**

**Name:** troponin C type 2 (fast)

**Aliases:** Troponin C, skeletal muscle

<http://www.ihop-net.org/UniPub/iHOP/mgi/92767.html>

**TRIM36**

**Name:** tripartite motif-containing 36

**Aliases:** HAPRIN, RBCC728, RING finger protein 98, RNF98, Tripartite motif-containing protein 36, Zinc-binding protein Rbcc728

<http://www.ihop-net.org/UniPub/iHOP/mgi/100607.html>

**TSC2**

**Name:** tuberous sclerosis 2

**Aliases:** FLJ43106, LAM, TSC4, tuberin, Tuberin, Tuberous sclerosis 2 protein

<http://www.ihop-net.org/UniPub/iHOP/mgi/92889.html>

**TSPO**

**Name:** translocator protein (18kDa)

**Aliases:** BZRP, DBI, IBP, MBR, mDRC, Mitochondrial benzodiazepine receptor, PBR, Peripheral-type benzodiazepine receptor, pk18, PKBS, PTBR, Translocator protein

<http://www.ihop-net.org/UniPub/iHOP/mgi/86810.html>

**TTN**

**Name:** titin

**Aliases:** CMD1G, CMH9, CMPD4, Connectin, CONNECTIN, DKFZp451N061, EOMFC, FLJ26020, FLJ26409, FLJ32040, FLJ34413, FLJ39564, FLJ43066, HMERF, LGMD2J, Rhabdomyosarcoma antigen MU-RMS-40.14, Titin, TMD

<http://www.ihop-net.org/UniPub/iHOP/mgi/92913.html>

**TTK**

**Name:** TTK protein kinase

**Aliases:** CT96, Dual specificity protein kinase TTK, ESK, FLJ38280, MPS1, MPS1L1, Phosphotyrosine picked threonine-protein kinase, PYT

<http://www.ihop-net.org/UniPub/iHOP/mgi/92912.html>

**USH1C**

**Name:** Usher syndrome 1C (autosomal recessive, severe)

**Aliases:** AIE75, AIE-75, Antigen NY-CO-38/NY-CO-37, Autoimmune enteropathy-related antigen AIE-75, DFNB18, harmonin, Harmonin, NY-CO-37, NY-CO-38, PDZ-45, PDZ73, PDZ-73, PDZ-73/NY-CO-38, PDZ-73 protein, Renal carcinoma antigen NY-REN-3, ush1cpst, Usher syndrome type-1C protein

<http://www.ihop-net.org/UniPub/iHOP/mgi/95222.html>

**VANGL1**

**Name:** vang-like 1 (van gogh, Drosophila)

**Aliases:** Loop-tail protein 2 homolog, LPP2, MGC5338, STB2, STBM2, Strabismus 2, Vang-like protein 1, Van Gogh-like protein 1

<http://www.ihop-net.org/UniPub/iHOP/mgi/103616.html>

**VSNL1**

**Name:** visinin-like 1

**Aliases:** Hippocalcin-like protein 3, HLP3, HPCAL3, HUVISL1, VILIP, VILIP-1, Visinin-like protein 1, VISL1, VLP-1

<http://www.ihop-net.org/UniPub/iHOP/mgi/93075.html>

**ZBTB20**

**Name:** zinc finger and BTB domain containing 20

**Aliases:** Dendritic-derived BTB/POZ zinc finger protein, DKFZp566F123, DPZF, HOF, ODA-8S, Zinc finger and BTB domain-containing protein 20, Zinc finger protein 288, ZNF288

<http://www.ihop-net.org/UniPub/iHOP/mgi/97423.html>
